# *Plasmodium falciparum* gametocytes display global chromatin remodelling during sexual differentiation

**DOI:** 10.1186/s12915-023-01568-4

**Published:** 2023-04-03

**Authors:** Myriam D. Jeninga, Jingyi Tang, Shamista A. Selvarajah, Alexander G. Maier, Michael F. Duffy, Michaela Petter

**Affiliations:** 1 Mikrobiologisches Institut – Klinische Mikrobiologie, Immunologie und Hygiene, Universitätsklinikum Erlangen, Friedrich-Alexander-Universität (FAU) Erlangen-Nürnberg, Erlangen, Germany; 2 Department of Medicine, University of Melbourne, Bio21 Institute, 30 Flemington Road, Parkville, VIC 3052 Australia; 3 The Australian National University, Research School of Biology, 134 Linnaeus Way, Canberra, ACT 2601 Australia; 4 Department of Microbiology and Immunology, University of Melbourne, Peter Doherty Institute, 792 Elizabeth Street, Melbourne, VIC 3000 Australia; 5 Bio21 Institute, 30 Flemington Road, Parkville, VIC, 3052 Australia

**Keywords:** *Plasmodium falciparum*, Malaria, Gametocytogenesis, Epigenome, Transcriptome, Histone variants

## Abstract

**Background:**

The protozoan malaria parasite *Plasmodium falciparum* has a complex life cycle during which it needs to differentiate into multiple morphologically distinct life forms. A key process for transmission of the disease is the development of male and female gametocytes in the human blood, yet the mechanisms determining sexual dimorphism in these haploid, genetically identical sexual precursor cells remain largely unknown. To understand the epigenetic program underlying the differentiation of male and female gametocytes, we separated the two sexual forms by flow cytometry and performed RNAseq as well as comprehensive ChIPseq profiling of several histone variants and modifications.

**Results:**

We show that in female gametocytes the chromatin landscape is globally remodelled with respect to genome-wide patterns and combinatorial usage of histone variants and histone modifications. We identified sex specific differences in heterochromatin distribution, implicating exported proteins and ncRNAs in sex determination. Specifically in female gametocytes, the histone variants H2A.Z/H2B.Z were highly enriched in H3K9me3-associated heterochromatin. H3K27ac occupancy correlated with stage-specific gene expression, but in contrast to asexual parasites this was unlinked to H3K4me3 co-occupancy at promoters in female gametocytes.

**Conclusions:**

Collectively, we defined novel combinatorial chromatin states differentially organising the genome in gametocytes and asexual parasites and unravelled fundamental, sex-specific differences in the epigenetic code. Our chromatin maps represent an important resource for future understanding of the mechanisms driving sexual differentiation in *P. falciparum.*

**Supplementary Information:**

The online version contains supplementary material available at 10.1186/s12915-023-01568-4.

## Background

Malaria poses a global health burden with more than 400,000 deaths each year, even though progress towards the elimination of malaria has been made in the past decades [1]. The *Plasmodium* parasites, the infectious agents causing malaria, have a complex life cycle, in which they undergo asexual replication in the human host and sexual replication in the Anopheles mosquito. For successful transmission from the human to the vector, the parasites need to differentiate into male and female gametocytes in a process referred to as gametocytogenesis. During this process that takes around 10 to 12 days in *Plasmodium falciparum*, the parasites sequester in the bone marrow [2, 3] and dramatically change their metabolism, transcriptome and morphology [4–6]. Upon maturation the gametocytes are released into the blood stream and can subsequently be taken up by the bite of a mosquito. Inside the midgut of the mosquito, the male gametocytes undergo three rounds of replication and exflagellate to form eight flagellated microgametes, while the female gametocytes round up and differentiate into macrogametes. Together the gametes then form the zygote, in which meiosis occurs and which differentiates into the motile ookinete that ultimately crosses the midgut wall of the mosquito [7]. On the other side in the hemocoel the ookinete forms an oocyst in which haploid sporozoites develop, which after maturation invade into the salivary glands for subsequent infections [8]. Therefore, the formation of male and female gametocytes is the key for transmission, and understanding more of the fundamental biology is required for transmission blocking strategies and ultimately malaria elimination [9].

Male and female gametocytes can be morphologically distinguished from each other around day seven of development, when they are classified as stage IV gametocytes. Female gametocytes are characterised by condensed hemozoin, an accumulation of osmiophilic bodies which are involved in gamete emergence, a high density of ribosomes and a relatively small nucleus with a nucleolus, consistent with their need to engage in protein synthesis important for the emerging macrogamete and zygote [10]. mRNAs coding for proteins important immediately post-fertilization are synthesized early and stored in ribonucleoprotein particles involving the RNA helicase DOZI [11]. By contrast, in the male gametocyte hemozoin appears more scattered and the nucleus is larger and apparently lacks a nucleolus, but kinetochores and microtubule organizing centres are present, which are required for rapid mitotic division after activation in the mosquito. Importantly, proteomic studies of separated male and female gametocytes in the rodent malaria parasite *P. berghei* and the human malaria parasite *P. falciparum* showed that the majority of gametocyte specific proteins are found pre-dominantly either in male or in female gametocytes, reflecting the fundamentally different biological functions of the two sexes [4, 12].

Little is known about how the sexual dimorphism in *P. falciparum* is regulated. It has been shown that epigenetic regulation plays a critical role for commitment to gametocytogenesis by de-repression of the transcription factor AP2-G, which is silenced by H3K9me3 and its associated factor heterochromatin protein 1 (HP1) in asexual parasites [13–16]. Furthermore, epigenetic mechanisms appear also important during gametocyte differentiation, as several epigenetic drugs (like histone deacetylase inhibitors or demethylase inhibitors) severely affect gametocytogenesis by transcriptional deregulation, and thereby prevent gametocyte maturation [17–21]. However, there is only limited data on the underlying chromatin landscape of gametocytes.

In asexual parasites, the chromatin is principally divided into the transcriptionally permissive euchromatin and the transcriptionally silent heterochromatin. The latter is marked by H3K9me3 and its associated protein HP1 and restricted to subtelomeric regions and internal islands, where it is involved in the repression of virulence-associated multigene families, such as genes of the *var* gene family encoding the major virulence factor PfEMP1 [22], and other exported proteins, that are expressed in a clonally variant way [23–28]. This heterochromatin compartment has been described as inherited over generations of asexually developing parasites [29]. Another histone modification present at heterochromatic loci is PfSET2/PfSETvs-mediated H3K36me3, which also contributes to *var* gene silencing [30].Conversely, the euchromatic compartment covers most of the genome and is marked by various other histone modifications and histone variants. The evolutionary conserved histone variant H2A.Z is associated with diverse functions. It has been shown to be important both for gene activation and silencing [31] and is implicated in embryonic stem cell differentiation and self-renewal by mediating access of activating and repressive complexes to regulatory regions [32]. In yeast, H2A.Z also has a boundary function to prevent heterochromatin spreading into euchromatic regions [33]. In asexual *Plasmodium* parasites H2A.Z occupies intergenic regions across the euchromatic genome irrespective of their activity, however H2A.Z enrichment levels positively correlate with transcription levels suggesting it may define promoter strength [34–36]. H2A.Z is usually absent from heterochromatic areas but is implicated in the regulation of *var* genes, as it is deposited in the promoter of the transcribed *var* gene together with the histone variant H2B.Z [35, 37]. The histone variant H2B.Z is specific for apicomplexan parasites [38] and in *P. falciparum* co-occurs in the same nucleosomes with H2A.Z throughout the genome [37, 39]. The histone modification H3K4me3 is enriched in promoter regions near the TSS in asexual parasites [23, 34, 35, 40, 41]. In other eukaryotes it is also located in the nucleosomes surrounding the TSS and contributes to transcription initiation, elongation and RNA processing and might play a role in recruiting histone deacetylases [42]. H3K4me3 often occurs in the same nucleosomes as the histone acetylation mark H3K9ac, which strongly correlates with transcription in *P. falciparum* [34]. In addition, H3K27ac is another histone acetylation associated with active gene expression in asexual parasite stages [36] and in murine embryonic stem cells, H3K27ac is enriched at active enhancer elements and implicated in cell differentiation by regulating cell type specific transcription programs [43]. Acetylation of H2A.Z, which also occurs in *Plasmodium* [44], also positively correlates with active transcription in metazoans [31] and is bound by several chromatin remodelling complexes in *P. falciparum* [45]. In the protozoan parasite *T. brucei*, acetylated H2A.Z is enriched at TSS nucleosomes of polycistronic transcription units where its presence correlates with gene expression, possibly by being involved in RNA PolII recruitment [46].

Few studies have addressed how chromatin structure impacts gene regulation during sexual development. It has been demonstrated using bulk gametocyte cultures that the transcriptome changes dynamically during the 10 day gametocyte maturation process [5, 47, 48] and this is accompanied by significant remodelling of the three dimensional genome organization in the nucleus of gametocytes, in particular with respect to the heterochromatin compartment [49]. Profiling of the heterochromatin landscape of immature and mature *P. falciparum* gametocytes characterised by H3K9me3 and HP1 indicated that several gametocyte specific genes as well as the erythrocyte remodelling machinery are differentially marked in gametocytes, implicating these processes in gametocyte maturation [49, 50]. Moreover, the repressive marks H3K36me2 and H3K36me3 have been linked to the regulation of a transcriptional shift in immature gametocytes by repressing genes relevant for asexual replication and the commitment factor AP2-G [51]. Importantly, in none of these studies male and female gametocytes were separated, despite the fundamentally different biology of these two cell types. The proteome and transcriptome of isolated mature male and female gametocytes has been profiled [4, 12, 52] and recent single cell RNAseq studies are beginning to shed light on sex-specific transcriptional differences during gametocytogenesis [53, 54]. However, thus far, only one study conducted in *P. berghei* investigated the differences of HP1 and H3K9ac coverage separately in mature male and female gametocytes, revealing differences in their heterochromatin and euchromatin landscape pointing to a complex epigenetic regulation underlying the different gene expression patterns observed [55]. In *P. falciparum*, no data is available on how the sex-specific transcriptomes change over gametocyte differentiation and how the chromatin landscape organises the genome to regulate sex-specific gene expression patterns.

To close some of the gaps in our knowledge of sexual differentiation in malaria parasites, we set out to study the transcriptome of separated male and female *P. falciparum* gametocytes during their maturation and to investigate the underlying epigenetic landscape. To this end we profiled several histone modifications using chromatin immunoprecipitation followed by high-throughput sequencing (ChIPseq) in asexual parasites, female gametocytes, and for some modifications also in male gametocytes, and correlated these with the transcriptional data. Finally, we also analysed the combinatorial patterns of these modifications in female gametocytes and ring stage parasites, generating comprehensive chromatin state maps for the two parasite stages.

## Results

### Separation of male and female gametocytes by FACS sorting

**Fig. 1:**
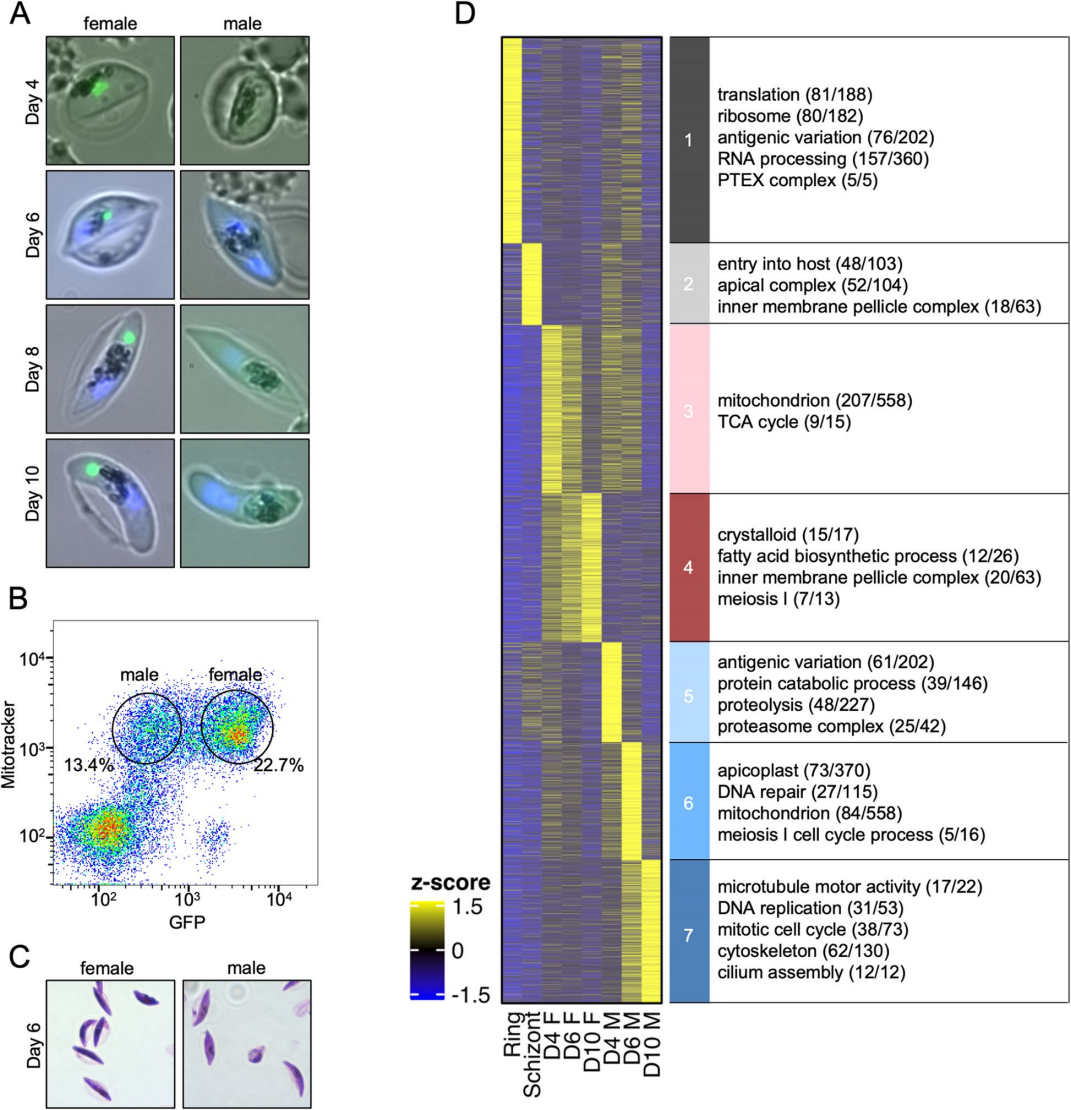
Gene expression profiling of asexual and sexual parasites. Parasites expressing GFP- tagged ABCG2 [56] were tightly synchronised and induced to generate gametocytes that were subsequently FACS-sorted to separate male and female gametocytes. **A** Validation of sex- specific GFP expression over the course of gametocytogenesis by fluorescence microscopy. GFP signal is shown in green, Hoechst33342 signal is shown in blue. **B** Gating strategy for FACS sorting of viable male (MitoTrackerTM Deep Red positive, GFP low) and female (MitoTrackerTM Deep Red positive, GFP positive) gametocytes. **C** Validation of morphology by Giemsa staining after sorting. **D** RNA of FACS sorted male (day 4: *n* = 3, day 6: *n* = 2, day 10: *n* = 1) and female gametocytes (day 4: *n* = 2, day 6: *n* = 4, day 10: *n* = 4) harvested on day 4, 6, or 10 of gametocytogenesis was sequenced along with RNA from ring (*n* = 2) and schizont stage (*n* = 1) parasites. The z-scored, k-means clustered heatmap indicates peak expression of sense transcripts in the different parasite stages. The most significant gene ontology (GO) terms are shown and gene numbers are indicated in brackets

To study the transcriptome and epigenome of male and female gametocytes, developing gametocytes were separated by FACS sorting using a sex-specific strategy. The previously described 3D7::ABCG2-GFP parasite line, which carries an endogenously GFP-tagged version of the female specifically expressed *ABCG2* gene was used [56]. These parasites express GFP from day 4 (stage II/III) onwards in female, but not in male gametocytes (Fig. 1A). To sort only viable gametocytes, parasites were stained with mitotracker dye, which relies on the mitochondrial membrane potential. Thus, mitotracker positive, GFP low parasites represent living male gametocytes and mitotracker positive, GFP positive parasites represent living female gametocytes (Fig. 1B). The morphology of the sorted cells was subsequently validated by Giemsa smears (Fig. 1C), which confirmed that male gametocytes develop slightly slower than female gametocytes as described before [57]. To rule out the possibility that parasites sorted as early male gametocytes based on the lack of GFP expression may be contaminated with early female parasites not yet expressing ABCG2-GFP, we cultivated a proportion of the parasites sorted on day 4 for two days and reanalysed them by FACS (Additional File 1: Figure S1A and B). This clearly demonstrated that GFP-negative male gametocytes sorted on day 4 remained GFP negative, whereas the GFP-positive female gametocyte population remained GFP-positive. After RNA sequencing of asexual ring and schizont stage parasites as well as the male and female gametocyte populations sorted on day 4 (stage II/III), 6 (stage III/IV) and 10 (stage V) of differentiation, their sex and stage were further validated by analysing the expression patterns of a panel of previously described genes known to be expressed in early gametocytes, mature male or female gametocytes, as well as some typical asexual marker genes (Additional File 1: Figure S1C) [4, 53, 57]. The expression pattern of the respective genes matched the predicted stage of the populations, showing that the sorting of male and female gametocytes was successful. Comparison of the global correlation of all expression datasets indicated that asexual parasites and gametocytes clearly clustered apart from each other, as did to a lesser extent the male and female gametocytes (Additional File 1: Figure S1D). Interestingly, the male gametocytes showed a higher transcriptional variability between the different time points (D4 vs D6 *R*^2^ = 0.83; D6 vs D10 *R*^2^ = 0.84; D4 vs D10 *R*^2^ = 0.64) than the female gametocytes (D4 vs D6 *R*^2^ = 0.98; D6 vs D10 *R*^2^ = 0.96; D4 vs D10 *R*^2^ = 0.93), suggesting that the male gametocytes transcriptome is more dynamic during sexual maturation (Additional File 1: Figure S1D). Of note, due to limited material recovered after FACS sorting, we were only able to successfully generate a sequencing library for a single D10 male replicate (Additional File 2: Table S1). However, a comparison to published mature male gametocyte transcriptome data demonstrated a significant correlation [4].

To identify genes with peak expression in different parasite stages, we used the z-scored sense transcript profiles of all genes over the stages studied and grouped them by k-means clustering. This yielded clusters of genes with highest expression in rings, schizonts, and three different maturation stages of male gametocytes, respectively, but only two groups for early and later female gametocytes (Fig. 1D). We analysed the genes in each cluster further for annotated gene ontology (Additional File 3: Table S2). The group of genes most highly expressed in ring stage parasites compared to other stages (Cluster 1) contained 1191 genes, of which 229 (19%) were of unknown function. The known genes were mainly implicated in “translation”, “ribosome biogenesis”, “mRNA binding / processing”, and contained most of the variant surface antigens. In contrast to that, 479 genes (121 (25%) of unknown function) with highest expression in schizonts (Cluster 2) were mostly invasion and egress related genes. This is consistent with stage specific transcriptional profiles from previous studies [58].

The genes with peak expression in female gametocytes fell into two clusters for early (day 4 and day 6) and later (day 6 and day 10) female gametocytes, respectively. The 969 genes (290 (30%) of unknown function) with peak expression in early female gametocytes (Cluster 3) contained mostly genes related to “mitochondrion”, which is maternally inherited during fertilisation [59], and “TCA cycle”, which reflects the metabolic changes from asexual parasites to gametocytes, where more glucose is catabolised in the TCA cycle and the parasites start to rely less on glycolysis alone [6]. In contrast, the 873 genes (288 (33%) of unknown function) with peak expression in later female gametocytes (Cluster 4), were mostly implicated in the formation of the inner membrane complex, which massively expands in gametocytes [60–62], and “meiosis I”, which is in accordance with preparation for meiosis in the zygote directly after fertilisation [63]. In addition, nearly all of the genes annotated to have a function in crystalloid formation (15 of 17), an ookinete specific transient organelle which is essential for sporogony [64], were most highly expressed in female gametocytes. We also found the female gametocyte transcription factor *ap2-fg* in this cluster, as well as *ap2-o*, which plays a role in the mosquito stages in rodent *Plasmodium* parasites [65, 66]. This is consistent with previous reports indicating that *ap2-o* transcripts are highly expressed but translationally repressed by DOZI in gametocytes [11].

In the male gametocytes, three clusters could be clearly distinguished (Cluster 5–7). Early male gametocytes (day 4 – stage II/III) showed highest expression of 579 genes (118 (20%) of unknown function). The predominant processes were related to proteolysis and proteasome complex, consistent with the critical function of proteostasis during metazoan spermatogenesis [67, 68]. In the intermediate male gametocytes (day 6 stage III/IV), 694 genes (224 (32%) of unknown function), were found to be highest expressed. Most were implicated in “apicoplast”, “DNA repair”, “mitochondrion”, and “meiosis I”. Finally, in mature male gametocytes (day 10 – stage V), the 844 peak expressed genes (318 (38%) of unknown function), were mainly related to “microtubule motor activity”, “DNA replication”, “mitotic cell cycle”, “cytoskeleton” and “cilium”, which is in agreement with preparation for male gametogenesis, where the activated male gametocytes undergo three rounds of DNA-replication, form axonemes and finally give rise to flagellated microgametes [63]. The expression of proteins linked to these pathways in mature male gametocytes is also supported by previous proteomic and transcriptomic results [4, 12, 69].

### The chromatin landscape differs globally between asexual parasites and female gametocytes

**Fig. 2:**
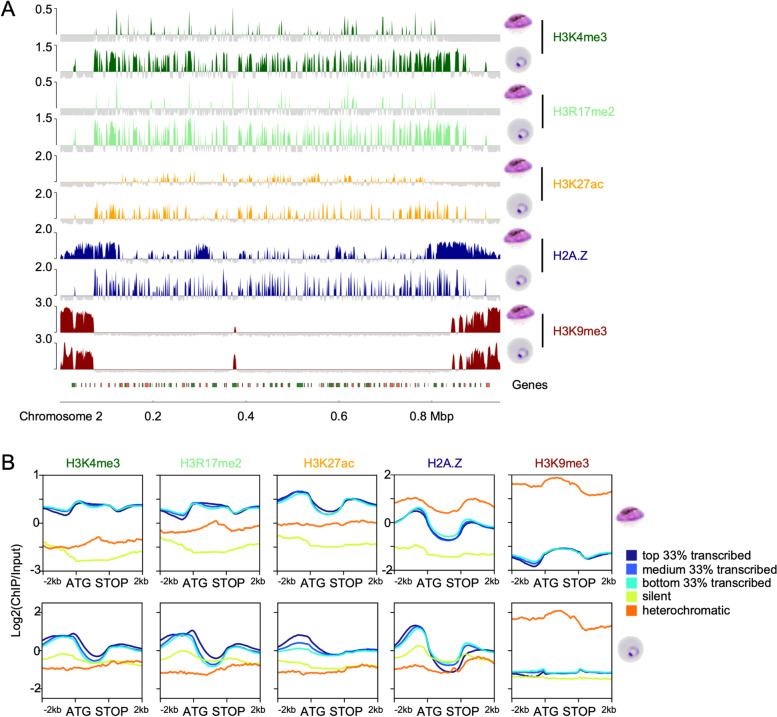
Global differences in the chromatin landscape between asexual parasites and gametocytes. ChIPseq was conducted on female day 6 gametocytes and ring stage parasites using antibodies against H3K4me3 (*n* = 2 for female gametocytes and ring stage parasites), H3R17me2 (*n* = 2 for female gametocytes and ring stage parasites), H3K27ac (*n* = 3 for female gametocytes, *n* = 2 for ring stage parasites), H2A.Z (*n* = 6 for female gametocytes, *n* = 2 for ring stage parasites), and H3K9me3 (*n* = 5 for female gametocytes, *n* = 2 for ring stage parasites). **A** Overview of log_2_-transformed ChIP/Input ratio tracks from female day 6 gametocytes and ring stage parasites along chromosome 2. Genes are indicated at the bottom, with green and orange referring to the transcriptional orientation. **B** Line plots showing the average log_2_-transformed ChIP/Input coverage of genes (ATG to STOP) and their 2 kb up- and downstream regions for each modification in female gametocytes (upper panel) and rings (lower panel) sorted into groups according to expression levels from stage-matched RNAseq data. Heterochromatic genes were defined by H3K9me3 enrichment 500 bp around the ATG. Silent genes were defined as genes with 0 FPKM by RNA-Seq. The remaining genes were divided into top, medium and bottom transcribed tertiles

It is known that the transcriptome of mature gametocytes is fundamentally different from asexual parasites [4, 5, 12] and that epigenetic mechanisms are involved in the commitment to gametocytogenesis [13–16, 70] and also play a critical role in the differentiation process [17–21, 44, 50]. To study the chromatin landscape of gametocytes underlying the observed transcriptional plasticity during differentiation, chromatin immunoprecipitation sequencing (ChIPseq) was conducted on female gametocytes and ring stage parasites. The female gametocytes were FACS sorted on day 6 based on GFP expression alone or on GFP and Mitotracker signal (Additional File 2: Table S1), and ChIPseq was performed for several euchromatin-associated modifications (H3K27ac, H3K4me3, H3R17me2, H2A.Z) as well as the heterochromatin marker H3K9me3. The presence of these modifications in gametocytes was confirmed using western blot (Additional File 1: Figure S2). For each modification, several biological replicates were included in the analysis as outlined in Additional File 2: Table S1. Figure 2A shows an overview of the enrichment profiles of all modifications over chromosome 2 in female gametocytes and ring stage parasites (all other chromosomes can be found in Additional File 1: Figure S3). The distinct patterns demonstrated that euchromatic histone marks are globally remodelled in female gametocytes, whereas H3K9me3 patterns remain relatively conserved. We wondered if this remodelling correlated with gene expression differences between the stages and therefore plotted the average coverage of the modifications over different gene groups sorted by expression level in rings and female gametocytes, respectively (Fig. 2B). We also defined a separate heterochromatic group of genes, which are covered by H3K9me3 for at least 500 bp around their ATG with a probability > 0.99999. The silent group consisted of genes that showed no transcription according to the RNAseq data, while the other three groups represent the terciles of all remaining genes sorted by expression value (FPKM). The line plots clearly show that the enrichment patterns change significantly between ring stage parasites and female gametocytes. For example, H3K4me3 and H3R17me2 are depleted from silent and heterochromatic genes in both, rings and gametocytes, but in expressed genes the enrichment is shifted from mainly upstream in rings to largely covering the gene body in female gametocytes, irrespective of the expression level (Fig. 2B). While H2A.Z is depleted from heterochromatin in rings, it showed a very strong enrichment in both subtelomeric and internal heterochromatin clusters in female gametocytes (Fig. 2B). These global differences were also obvious when plotting the gene groups and their coverage as heatmaps (Additional File 1: Figure S4A and C). In the following paragraphs, we will discuss the single modifications in more detail.

### H3K4me3 and H3R17me2 are depleted from promoters and enriched in gene bodies in female gametocytes

Tri-methylation of histone 3 lysine 4 (H3K4me3) is well-studied in asexual parasites, and is typically associated with promoters [34, 35]. Di-methylation of histone 3 arginine 17 (H3R17me2) has been detected in *Plasmodium* [44] but has not been functionally investigated yet. We included this modification in our study because in *Toxoplasma gondii*, TgCARM1 mediated H3R17me2 is implicated in parasite differentiation and is located in promoters of stage specifically expressed genes [71].

**Fig. 3:**
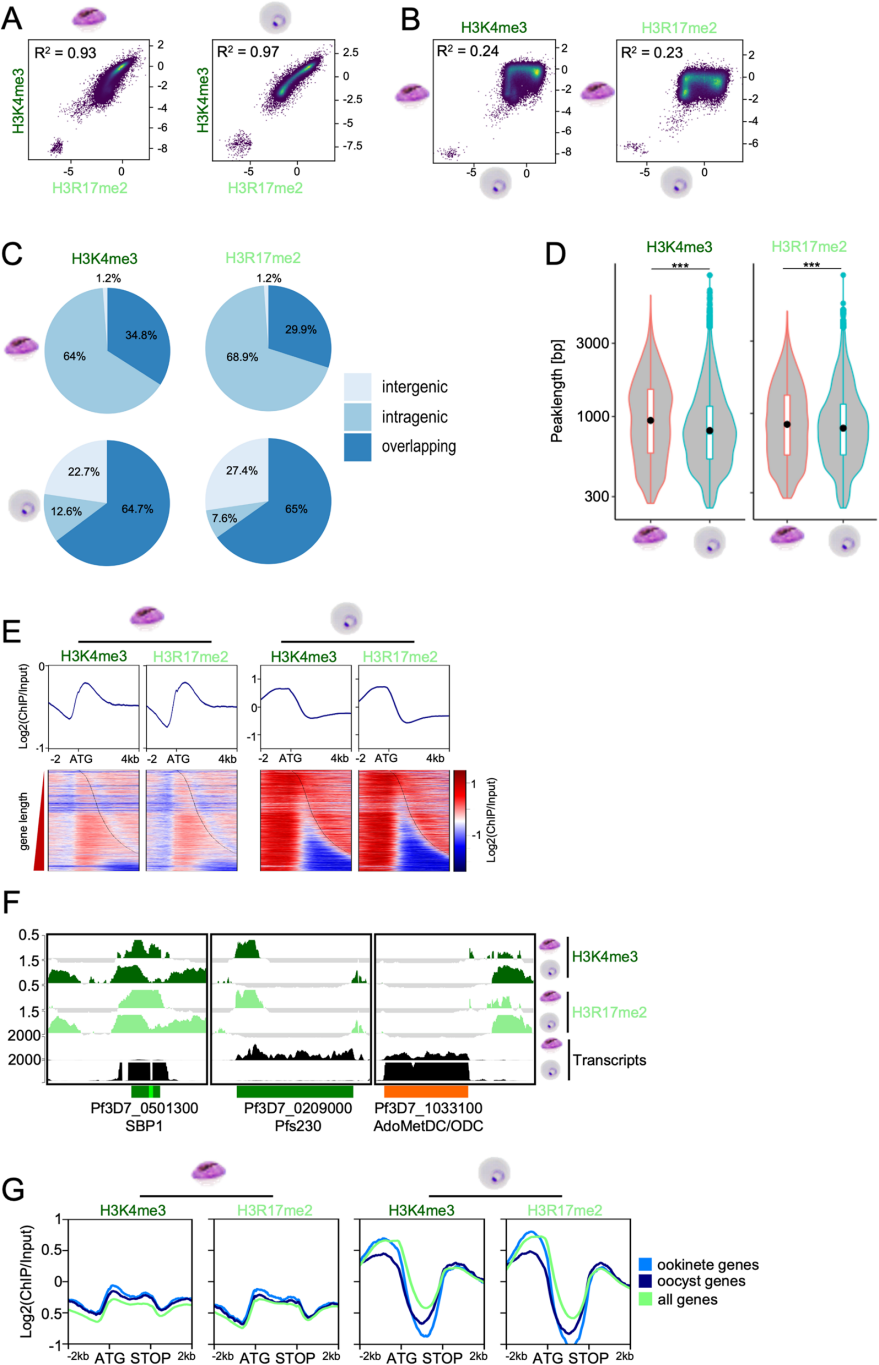
H3K4me3 and H3R17me2 overlap genome-wide and are shifted into gene bodies in gametocytes. **A** Scatter plots showing the spearman correlation of mean H3K4me3 versus H3R17me2 coverage from two biological replicates in female day 6 gametocytes and ring stage parasites. **B** mean H3K4me3 or H3R17me2 coverage from two biological replicates in gametocytes versus ring stage parasites, respectively. The genome was divided into 150 bins and the average log_2_-transformed ChIP/Input ratio was calculated. R^2^, spearman correlation coefficient. **C** Piechart showing percentages of peaks intersecting intergenic regions, intragenic regions, or both (overlapping). **D** Violin plot of peak lengths. Peaks were called by MACS2 in female gametocytes and ring stage parasites. A two-sided student’s t-test showed for H3K4me3 peaks test statistic t = 17.505, degrees of freedom df = 19,404, *p*-value < 2.2e-16; and for H3R17me2 peaks test statistic t = 6.3903, degrees of freedom df = 17,222, *p*-value = 1.698e-10. **E** Profile plots and heatmap of log_2_-tranformed ChIP/Input data from 2 kb upstream to 4 kb downstream of every gene centred on the ATG and sorted by gene length. The dashed line shows the STOP site of the respective genes. **F** Examples of H3K4me3 or H3R17me2 ChIPseq profiles for a selection of loci with differential peaks in female gametocytes and rings. Transcripts detected by RNA-Seq are shown in the bottom line (FPKM). SBP1, skeleton binding protein (ring specific expression), region shown from 65.5 kbp to 72 kbp; Pfs230, 6-cysteine protein P230 (gametocyte specific expression), region shown from 368.4 kbp to 381.16 kbp; AdoMetDC/ODC, S-adenosylmethionine decarboxylase/ornithine decarboxylase (upstream peaks in both stages), region shown from 1.324 Mbp to 1.332 Mbp. Genes are marked by orientation either green (+ genes) or orange (- genes), with introns marked in either light green or dark red. **G** Average log_2_-transformed ChIP/Input coverage plots for H3K4me3 and H3R17me2 in day 6 female gametocytes and ring stage parasites over ookinete specifically expressed genes (defined by [48], available on PlasmoDB), oocyst specific genes (compared to blood stage genes, defined by [74], available on PlasmoDB), and all genes

The global profile maps indicated that the patterns of H3K4me3 and H3R17me2 were nearly identical, both in gametocytes as well as in asexual parasites (Fig. 2). Indeed, no significant differences were found using the CSAW package, which is a tool used to uncover differential enrichment of histone modifications [72]. The peaks identified by MACS2 [73] demonstrated overlaps between 86 and 96%, which was similar to the variability between replicates of each modification. A scatter plot comparing the enrichment of the two modifications along 150 bp bins showed high correlation in both stages (*R*^2^ = 0.93 and *R*^2^ = 0.97 in female day 6 gametocytes and ring stage parasites, respectively), suggesting that H3K4me3 and H3R17me2 globally co-occur (Fig. 3A). In contrast, when analysing the correlation of H3K4me3 and H3R17me2 between female day 6 gametocytes and ring stage parasites, there was very low correlation (*R*^2^ = 0.24 for H3K4me3 and *R*^2^ = 0.23 for H3R17me2), indicating global remodelling of the H3K4me3/H3R17me2 modification landscape in sexual stages (Fig. 3B).

To further analyse the different distributions of H3K4me3 and H3R17me2 over the genomes of female gametocytes and ring stage parasites, we intersected MACS2 called peaks with either intergenic regions or coding sequences (intragenic). Peaks that would intersect both features were called “overlapping” peaks. In asexual parasites, most peaks were found to be overlapping (64.7% for H3K4me3, 65% for H3R17me2), or located in intergenic regions (22.7% for H3K4me3, 27.4% for H3R17me2), with a smaller proportion localising in coding regions (12.6% for H3K4me3, 7.6% for H3R17me2). In contrast in gametocytes the majority of peaks were intragenic (64% for H3K4me3, 68.9% for H3R17me2) or overlapping (34.8% for H3K4me3, 29.9% for H3R17me2), with only 1.2% accumulating in intergenic regions (Fig. 3C), indicating a global shift of H3K4me3 and H3R17me2 towards coding regions. In addition, we also observed that peaks in female gametocytes were significantly broader than in ring stage parasites (t test both *p* < 1.7e-10, Fig. 3D), which was particularly interesting because global repositioning of H3K4me3 into broad domains partially covering coding regions has been reported to be important for cell identity in mouse oocytes [75, 76]. When we plotted the histone modification coverage of all genes sorted by length centred on the ATG, both modifications clearly accumulated upstream of the ATG in ring stage parasites, whereas in female day 6 gametocytes they were depleted from this area and peaked downstream of the ATG instead (Fig. 3E). To further inspect how the local enrichment patterns of the two modifications correlate with gene expression, we visualised the coverage track in a selection of genes with different transcription profiles (Fig. 3F). The ring-specifically expressed gene *SBP1* showed peaks overlapping intra- and intergenic regions in both stages, but the peak was clearly shifted into the gene body and covering the entire open reading frame in female gametocytes (Fig. 3F, Additional File 1: Figure S5), whereas the ring stage parasites had an additional upstream peak, which was also enriched in H3K27ac (Additional File 1: Figure S5). In contrast, the gametocyte specific gene *Pfs230* had only marginal enrichment of H3K4me3/H3R17me2 in its upstream region in ring stage parasites but showed a strong coverage accumulating at the gene start in female gametocytes (Fig. 3F, Additional File 1: Figure S5). This was similar in the gametocyte specifically expressed gene *PUF1*, which also showed enrichment near the ATG in gametocytes but in contrast to *Pfs230* additionally had a strong upstream peak of H3K4me3/H3R17me2 in rings (Additional File 1: Figure S5). The enrichment near the ATG was reminiscent of the ATG peak in the most strongly expressed genes in ring stage parasites (Fig. 2B, Additional File 1: Figure S4C) and suggests that H3K4me3 and H3R17me2 positioning rather than presence is important for gene transcription, as was also reported for asexual parasites [41] (Fig. 2B). Only a few peaks were located exclusively in the intergenic region, one example is upstream of *AdoMetDC/ODC* (S-adenosylmethionine decarboxylase/ornithine decarboxylase), which has upstream peaks in ring stage parasites as well as female day 6 gametocytes and is expressed in both stages (Fig. 3F). However, there was generally no obvious correlation of the H3K4me3/H3R17me2 enrichment pattern with gene expression in female gametocytes (Fig. 2B).

It has been described previously that asexual parasites show stage specific differences in the deposition of H3K4me3 [34], so we separately plotted H3K4me3 and H3R17me2 profiles over genes with peak expression in female gametocyte and rings. This showed slightly increased levels of the methyl marks upstream of female specific genes in female gametocytes (Additional File 1: Figure S6). To test whether H3K4me3 might broadly mark genes of functional importance after fertilisation, as reported during maternal-to-zygote transition [75, 76], we analysed genes that are specifically transcribed in ookinetes [48] and genes that are upregulated in oocyst in comparison to blood stage parasites [74]. Indeed, we observed significantly higher enrichment of H3K4me3 in the upstream region and in the gene body of both, ookinete and oocyst expressed genes in female day 6 gametocytes compared to the average of all genes, and particularly around the ATG (Fig. 3G, Additional File 1: Figure S7). In contrast, in ring stage parasites there were slightly lower levels of H3K4me3/H3R17me2 over ookinete specific gene bodies, and a significant reduction across oocyst specific genes compared to the average of all genes (Fig. 3G, Additional File 1: Figure S7). It was recently reported that treatment of trophozoites with the Jmj histone demethylase inhibitor JIB-04 induces histone hypermethylation and leads to upregulation of genes functionally important in ookinetes [18]. Indeed, in rings these genes carried lower levels of H3K4me3 and H3R17me2 in comparison to all genes, consistent with active histone de-methylation at these loci. However, in female gametocytes we detected a similar enrichment of H3K4me3/H3R17me2 in this JIB-04 upregulated ookinete gene set as in all ookinete genes (Additional File 1: Figure S7). Together, these observations hint to a role of H3K4me3/H3R17me2 in poising genes in female gametocytes that are functionally important after fertilization.

### H3K9me3 shows sex specific differences between male and female gametocytes

**Fig. 4:**
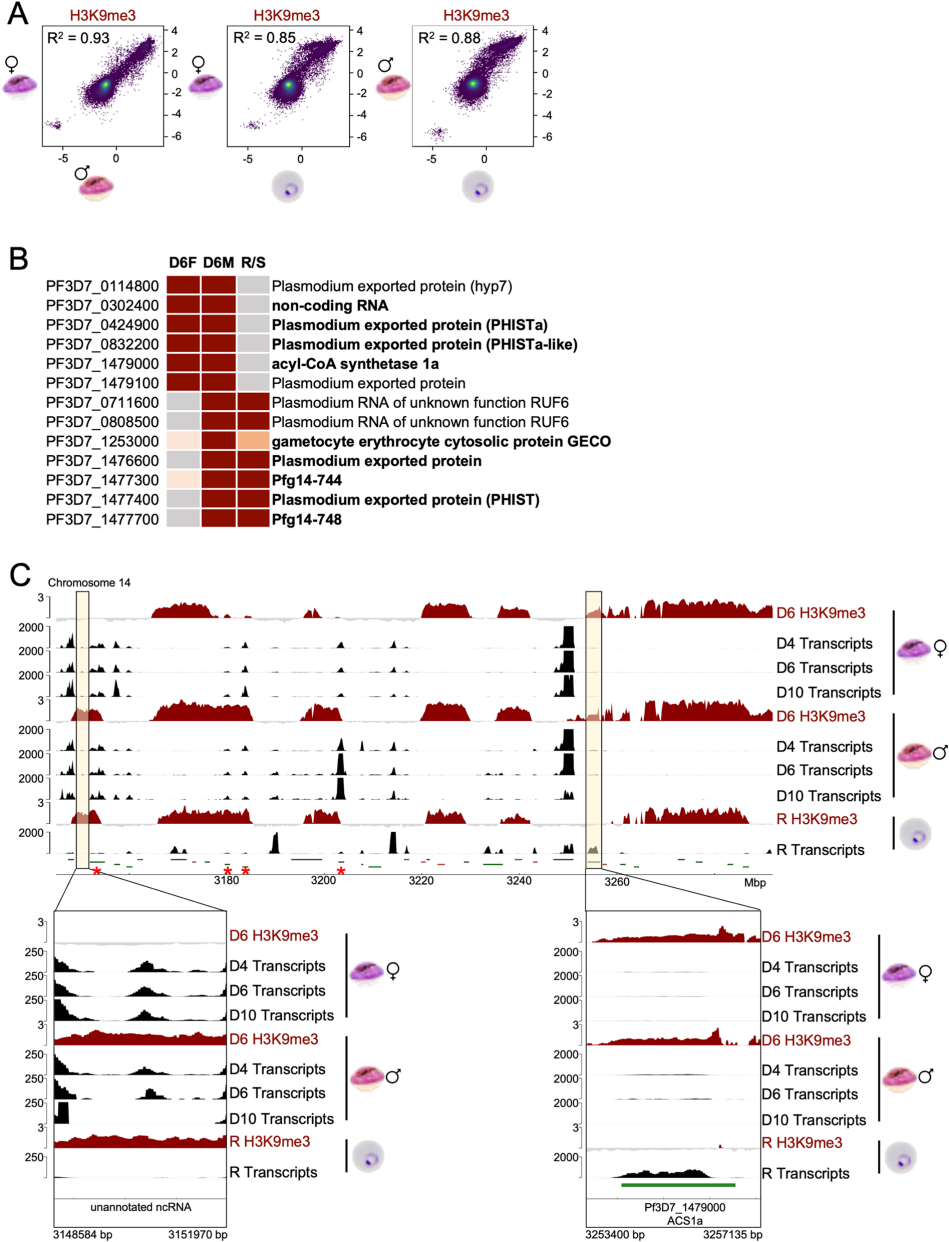
Differences in H3K9me3-associated heterochromatin between immature female and male gametocytes and ring stage parasites. **A** Scatter plot shows pearson correlation of mean H3K9me3 coverage per 150 bp bin between the three parasite stages (*n* = 5 for female gametocytes, *n* = 2 for male gametocytes, *n* = 2 for ring stage parasites). R^2^, pearson correlation coefficient. **B** Table of genes marked differentially with H3K9me3 in the three stages. Red = H3K9me3, grey = no H3K9me3, orange = partial H3K9me3 (in promoter region but not in the gene body), light orange = reduced coverage with H3K9me3, D6M = male day 6 gametocyte, D6F = female day 6 gametocyte, R/S = rings and schizonts. **C** Overview over transcription profiles (black) and log_2_-transformed ChIP/Input ratio tracks of H3K9me3 (dark red) covering the right end of chromosome 14 to depict differences in the subtelomeric region between male and female gametocytes and rings. RNAseq data are shown for males and females from day 4, day 6, and day 10 during gametocytogenesis. The enlargement highlights two regions that are differentially marked: a putative lncRNA identified between Pf3D7_1476500 and Pf3D7_1476600 (left), and acyl-CoA synthetase ACS1a (right). H3K9me3 coverage of the lncRNA locus in rings and day 6 male gametocytes correlates with suppressed transcription in rings and day 10 male gametocytes. H3K9me3 coverage of ACS1a in male and female gametocytes correlates with suppressed transcription throughout gametocyte differentiation

H3K9me3 and HP1 are markers of heterochromatin in *Plasmodium* parasites [23–25, 27] and their genome-wide distribution is largely preserved between asexual parasites and mature gametocytes, except for some areas that in particular involve exported gene families and host cell remodelling factors [49, 50]. As H3K9me3 was described to play a role in determination of the cell fate in mammalian cells [77], we were particularly interested to identify differentially marked genes between male and female gametocytes and asexual parasites. Thus, we performed ChIPseq for H3K9me3 in all of these stages (Fig. 4).

Generally, H3K9me3 was highly conserved at telomeres and subtelomeres and over intrachromosomal islands covering variant surface antigen genes as described previously [23–25, 49, 50, 55]. Indeed, pearson correlation coefficients were high between the parasite stages (*R*^2^ = 0.93 between male and female gametocytes and *R*^2^ = 0.85 and *R*^2^ = 0.88 between female or male gametocytes and ring stage parasites, respectively) (Fig. 4A). However, some differential coverage could be observed between rings, male gametocytes and female gametocytes (Fig. 4B), especially on the right arm of chromosome 14 (Fig. 4C). Intriguingly, we identified a region that was H3K9me3 covered in rings and male gametocytes but not in female gametocytes (Fig. 4C, left panel). Inspection of the RNA profile in this area suggested that this region may represent a differentially regulated non-coding RNA (ncRNA) in the 5’ region of Pf3D7_1476600, as we observed transcripts specifically in female gametocytes and early male gametocytes, but not in mature male gametocytes and rings. This is consistent with heterochromatin silencing of this transcript in mature males and asexual parasites, indicating a sex specific function warranting further examination. Another non-coding RNA (Pf3D7_0302400), which was described to be involved in the regulation of clag3 expression [78], was covered with H3K9me3 in gametocytes but not in ring stage parasites (Fig. 4B). This was similar for the gene coding for acyl-CoA synthetase ACS1a (Pf3D7_1479000), which is located near the heterochromatin boundary on the right side of chromosome 14 and is transcribed only in ring stages but not in gametocytes, consistent with its heterochromatin mediated silencing during sexual differentiation (Fig. 4C, right panel). In this region on chromosome 14 there are also several early gametocyte marker genes located (Pf3D7_1476600, Pf3D7_1477300 (Pfg14-744), Pf3D7_1477400, Pf3D7_1477700 (*Pfg14-748*), Pf3D7_1478000 (*gexp17*)) [79], which have been described to be differentially enriched with either H3K9me3 or HP1 between mature gametocytes and asexual parasites [49, 50]. Of those loci, we found the exported protein Pf3D7_1476600, and the PHIST protein coding genes Pf3D7_1477400 and Pf3D7_1477700 uncovered from heterochromatin in female gametocytes but not in male gametocytes (Fig. 4B). Differential coverage was also observed for the PHIST protein coding gene Pf3D7_1477300, with partial H3K9me3 depletion in female gametocytes. The gene coding for the HSP40 paralog gametocyte erythrocyte cytosolic protein (GECO, Pf3D7_1253000) [80], on the other hand, was barely covered in female gametocytes but strongly in male gametocytes in its gene body and promoter region, whereas in ring stage parasites, only its promoter region was covered (Additional File 1: Figure S8A). Interestingly, transcription of *geco* did not correlate with H3K9me3 patterns, as highest transcription was observed in ring stage parasites, followed by male and then female gametocytes. As *geco* transcription was reported to peak in rings and early gametocytes [80], this may reflect the presence of some younger gametocyte stages in the ring population. On the other hand, the lower coverage in the female gametocytes may facilitate upregulation of *geco* expression later during the life cycle in salivary gland sporozoites [74].

Consistent with a function during commitment but not during gametocyte maturation, the gene coding for the sex-specific transcription factor AP2-G was covered with H3K9me3 in all stages. However, in gametocytes only the gene body was heterochromatic, whereas in ring stage parasites H3K9me3 stretched over the upstream region as well (Additional File 1: Figure S5, panel 2). This is in line with the previous observation that de-methylation of heterochromatin in the upstream region of the *ap2-g* gene is sufficient for its activation [81]. In female gametocytes, the upstream region was moreover enriched in H2A.Z, whereas H2A.Z was absent in rings (Additional File 1: Figure S5, panel 2). In conclusion, we identified sex specific patterns of heterochromatin, which could be of functional importance for the cellular identity of male and female gametocytes. A comparison of differentially heterochromatic genes identified in previous studies [49, 50] and our study is provided in Additional File 4: Table S3.

### H2A.Z is specifically enriched in heterochromatin in female gametocytes and co-occurs with H2B.Z

**Fig. 5:**
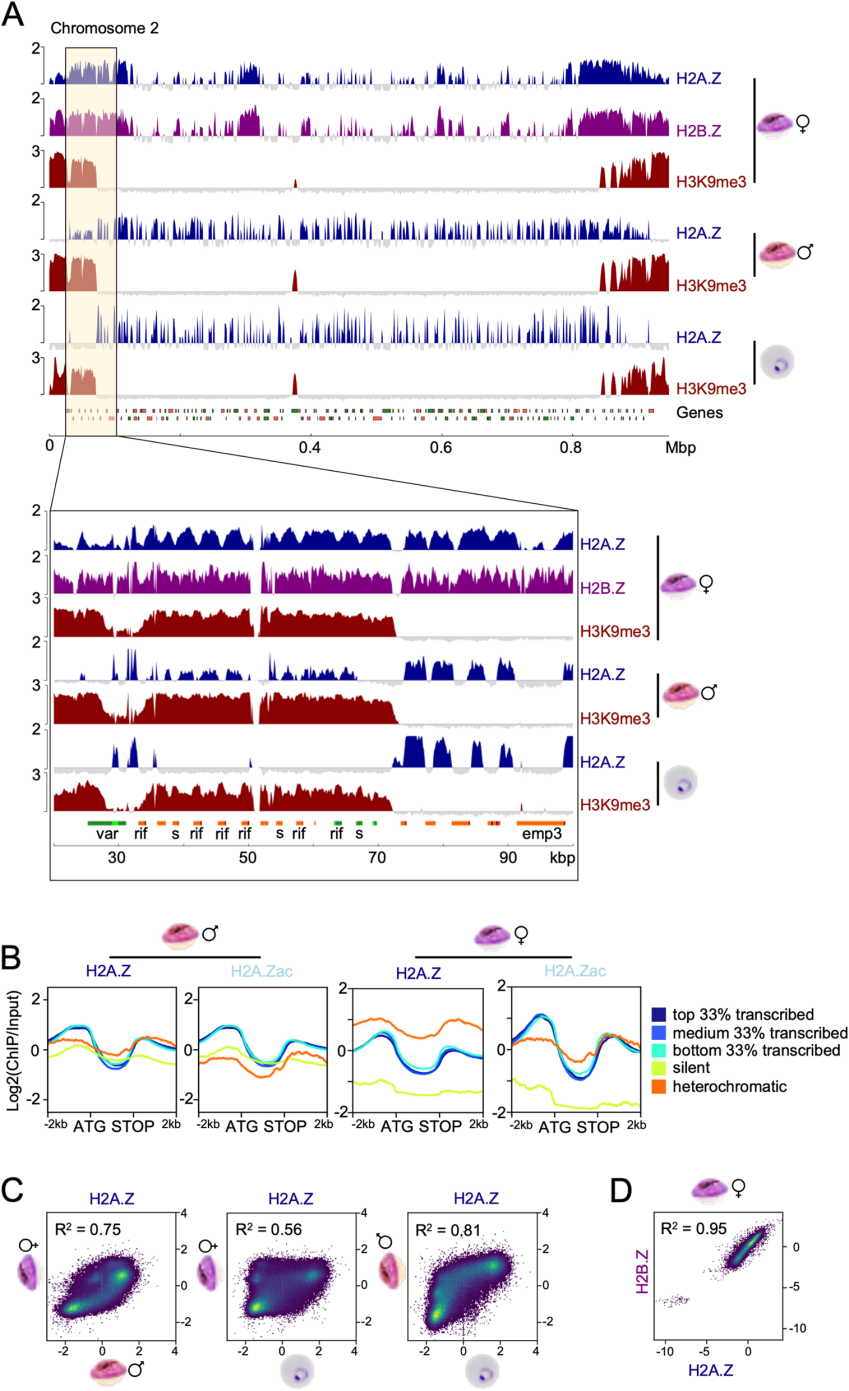
H2A.Z is associated with heterochromatic areas in day 6 female gametocytes. **A** Overview over chromosome 2 showing log_2_-transformed ChIP/Input coverage for H2A.Z (deep blue) (*n* = 6 for female gametocytes, *n* = 2 for male gametocytes, *n* = 2 for ring stage parasites) and H3K9me3 (deep red) (*n* = 5 for female gametocytes, *n* = 2 for male gametocytes, *n* = 2 for ring stage parasites) in rings, male and female gametocytes as well as H2B.Z (purple) in female gametocytes (*n* = 1). Lower panel shows zoom into the heterochromatin/euchromatin boundary in the left subtelomeric region of chromosome 2, which includes several *var, stevor* (marked with an “s”)*,* and *rif* genes. Genes are marked by orientation either green (+ genes) or orange (- genes), with introns marked in either light green or dark red. **B** Average profile plots of log_2_-transformed ChIP/Input signals for H2A.Z and H2A.Zac over gene bodies (ATG to STOP) and their 2 kb up-/downstream region in male and female day 6 gametocytes grouped by gene expression. **C** Correlation scatter plot of log_2_-tranformed ChIP/Input H2A.Z coverage per 150 bp bin comparing the different parasite stages against each other. R^2^, spearman correlation. **D** Correlation scatter plot of H2A.Z versus H2B.Z in female day 6 gametocytes

H2A.Z has been described to mark intergenic regions in *Plasmodium* where its levels correlate with transcriptional activity [34, 35]. Consistent with this, in ring stage parasites H2A.Z occupancy was strongest in the promoters of transcribed gene groups and significantly reduced in silent genes and heterochromatin (Fig. 2B). In female gametocytes, enrichment of H2A.Z in promoters of transcribed genes relative to silent genes was also evident; however, H2A.Z showed even stronger enrichment in heterochromatic regions (Fig. 2B). To determine whether this accumulation was female gametocyte specific or a feature of both sexes, we also performed ChIPseq of H2A.Z in male gametocytes (Fig. 5).

Inspection of the global pattern of H2A.Z, as shown for chromosome 2, revealed that H2A.Z accumulation in the intergenic regions of heterochromatic genes was much stronger in female gametocytes than in male gametocytes, although low levels of H2A.Z were detectable along subtelomeric and internal heterochromatin clusters in male gametocytes as well (Fig. 5A, Additional File 1: Figure S9). This difference is also evident from the coverage plots of H2A.Z over differentially expressed gene groups (Figs. 2B and 5B). Generally, H2A.Z coverage in day 6 male gametocytes resembled most that in ring stages with a correlation coefficient of 0.81, compared to a correlation coefficient of only 0.56 between ring stage parasites and female day 6 gametocytes, with clear enrichment of H2A.Z in heterochromatic regions in female gametocytes only (Fig. 5C). A higher correlation of 0.75 was calculated between male and female day 6 gametocytes, but again with a small cluster of bins away from the others, depicting the differences in H2A.Z heterochromatin coverage between male and female gametocytes.

In asexual parasites, H2A.Z is constitutively associated with the histone variant H2B.Z [37, 39], so we asked whether the association of the two histone variants was still maintained in the heterochromatin compartment in female gametocytes and performed ChIPseq using H2B.Z-specific antibodies. H2A.Z and H2B.Z clearly co-localised (Fig. 5A, Additional File 1: Figure S9) with a spearman correlation coefficient of 0.95 for 150 bp bins (Fig. 5D). The correlation was confirmed by the overlap of 91.6% of significant H2A.Z and H2B.Z peaks called by MACS2 in female gametocytes. Therefore, we conclude that the co-occupancy of the two histone variants is maintained genome-wide in female gametocytes. We further wondered whether H2A.Z in the heterochromatin compartment was acetylated and conducted ChIPseq using antibodies specifically recognizing H2A.Z acetylated at lysine 11 and 15 (Azizan et al., manuscript in preparation). In both, male and female gametocytes, heterochromatic genes showed very low levels of H2A.Zac as opposed to transcribed genes (Fig. 5B), indicating that the heterochromatin-associated H2A.Z is mostly not acetylated. This suggests that post-translational modifications of H2A.Z define its differential enrichment in eu- and heterochromatin in *P. falciparum* gametocytes.

In summary, we observed a strong, female gametocyte specific association of unacetylated H2A.Z and H2B.Z with subtelomeric as well as central heterochromatin clusters. H2A.Z/H2B.Z nucleosomes were particularly enriched in the intergenic regions of all heterochromatin associated gene families as well as in AP2-G, although an increased accumulation along gene bodies was also evident relative to euchromatic genes (Figs. 2B and 5B, Additional File 1: Figure S9).

### Histone acetylation enrichment in promoters correlates with stage specific gene expression in asexual and sexual parasites

After identifying global, sex specific remodelling of H3K4me3 and H2A.Z in gametocytes, we wondered whether acetylations of H3 and H2A.Z were also enriched in non-canonical patterns during gametocytogenesis. Both modifications strongly correlate with gene expression in various organisms [31, 46, 82–84] as do H3 acetylations in asexual *Plasmodium* parasites [36].

**Fig. 6:**
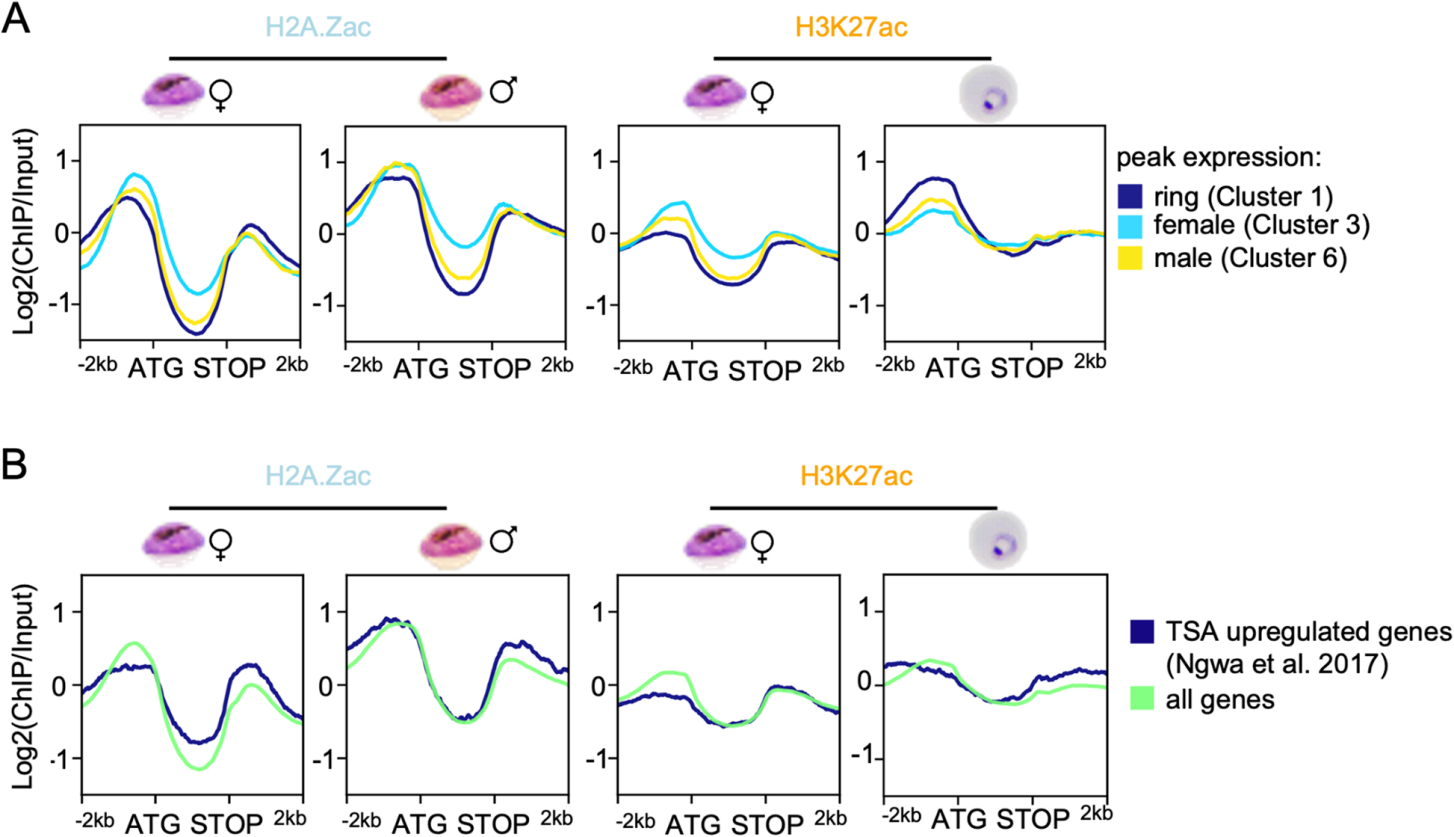
Histone acetylation H3K27ac and H2A.Zac correlate with stage specific gene expression in ring stage parasites and female gametocytes. **A** Average profile plots of log_2_-transformed ChIP/Input signals from H2A.Zac (*n* = 2 for female gametocytes, *n* = 1 for male gametocytes) and H3K27ac (*n* = 3 for female gametocytes) in female and male day 6 gametocytes for stage specifically expressed genes. Ring, female, and male specific genes represent clusters 1, 3, and 5, respectively, from Additional File 1: Figure S1. **B** Average log_2_-transformed ChIP/Input coverage plots for H2A.Zac and H3K27ac in day 6 male and female gametocytes over genes that are upregulated after TSA treatment in early gametocytes (Ngwa et al. 2017, available as Supplementary Table 2), and all genes

Consistent with a function in gene activation, in ring stage parasites H3K27ac was strongly enriched in the 5’ region of euchromatic genes and positively correlated with the transcription level (Fig. 2B, Additional File 1: Figure S4). However, in female gametocytes all transcribed genes showed a H3K27ac peak of similar magnitude in their 5’ region, regardless of their transcription level (Fig. 2B). This was also observed for H2A.Zac in male and female gametocytes (Figs. 2B and 5B). *Plasmodium* gametocytes have been reported to express and translationally repress many transcripts, which are relevant after fertilization [11, 85, 86]. As our matched RNA samples represented steady-state RNA rather than de novo synthesised RNA, the association of histone acetylation with transcription levels may be biased by the presence of stable, translationally repressed transcripts. Therefore, we excluded genes described to be translationally repressed in *P. falciparum* gametocytes [4] as well as orthologs of the genes described to be DOZI-stored in *P. berghei* [11, 86] from the list of top transcribed genes. However, there was no difference in the correlation of coverage with transcript levels (Additional File 1: Figure S10). To understand if histone acetylation was particularly relevant for genes that were regulated in a stage specific manner as described previously in asexual parasites [36], we plotted the enrichment profiles for the gene clusters that were differentially expressed between asexual parasites, female gametocytes and male gametocytes (Fig. 1). This approach revealed a stronger enrichment of H3K27ac in promoter regions of genes expressed predominantly in female gametocytes and in ring stage parasites in the respective stage. A similar trend was also evident for H2A.Zac in female gametocytes, albeit to a much lesser extent (Fig. 6A). An example for stage specific histone acetylation is *sbp1*, which is highly expressed in ring stage parasites and carries H3K27ac in its upstream region in ring stage but not in gametocytes (Additional File 1: Figure S5). Conversely, the gametocyte specifically expressed gene *pfs230* carries H3K27ac in its upstream region only in female gametocytes but not in ring stage parasites. In male gametocytes, H2A.Zac was only marginally more enriched in gametocyte specific genes compared to ring specific genes, and not in a sex-specific fashion (Fig. 6A).

The importance of correct histone acetylation for gametocytogenesis has been demonstrated in experiments with the histone deacetylase inhibitor TSA, which leads to hyperacetylation and gene deregulation in a subset of genes [21]. When we looked at the acetylation status of the genes upregulated by TSA treatment of immature gametocytes, we could indeed observe lower H2A.Zac and H3K27ac levels in the 5’ region in female gametocytes compared to all genes, indicating that these genes are actively suppressed by deacetylation in female gametocytes (Fig. 6B). Interestingly, there was only a marginal difference in this gene set in male gametocytes or ring stage parasites. In conclusion, histone acetylation, in particular H3K27ac, positively correlated with stage specific transcription in rings and female gametocytes, implicating it in the stage specific developmental program.

### Putting the epigenome on a map

**Fig. 7:**
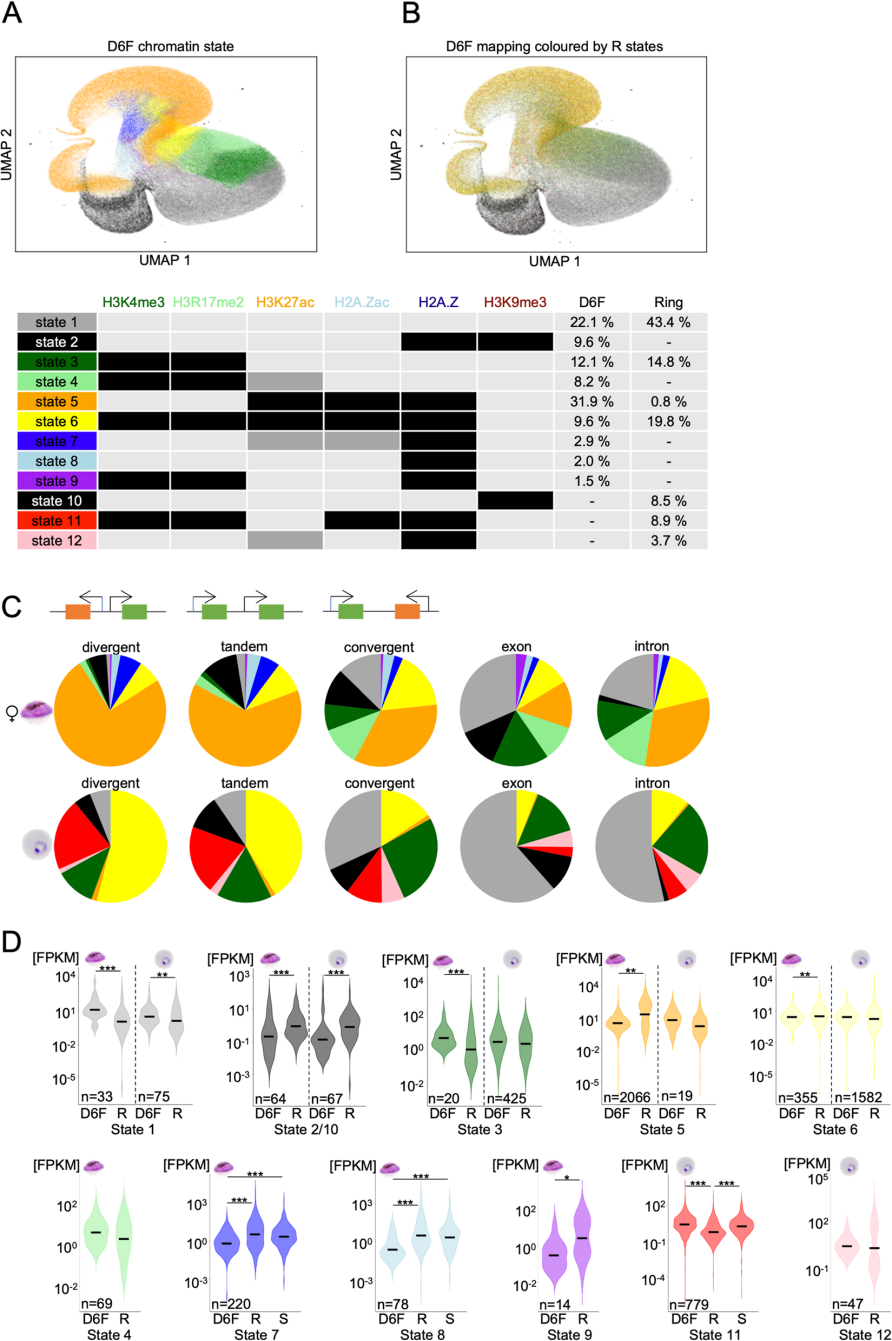
UMAP of the chromatin landscape defines chromatin states. **A** UMAP of the chromatin of female day 6 gametocytes, divided in bins of 50 bp, coloured by their individual chromatin states. **B** UMAP of female day 6 gametocytes coloured by ring stage chromatin states, showing conserved and remodelled bins. **C** Pie charts of the distribution of chromatin states over different genetic elements per parasite stage. **D** Violin plots showing the FPKM values of the genes that carried a region of at least 300 bp of the different states in their 2 kb upstream region in female day 6 gametocytes and ring stage parasites, respectively, indicated by the parasite stage above the plot. The FPKM values are compared between gametocyte gene expression (D6F), ring stage gene expression (R) and schizont stage gene expression (S) on the x-axis. Differences between the groups were investigated using Wilcoxon rank sum test with continuity correction (* *p* < 0.05, ** *p* < 0.01, *** *p* < 0.001). The black line represents the mean FPKM value

While we made very interesting and novel observations when analysing each modification individually, we strived to understand how the combinatorial pattern of histone modifications and histone variants globally shapes the genome of asexual and sexual malaria parasites. Therefore, we combined the ChIPseq data for all analysed histone modifications into multidimensional matrices for female gametocytes and ring stage parasites, which can be depicted using a uniform manifold approximation and projection (UMAP) (Fig. 7A). To do so, the genome was split into bins of 50 bp and the average coverage of each histone modification or variant was calculated per bin per stage. The resulting matrix of each parasite stage was then PCA reduced to two dimensions and clustered using the Leiden algorithm. The resulting clusters were correlated with the coverage values and either subclustered using k-means clustering if the cluster was ambiguous, or fused if clusters were similar to reduce the total number of clusters. The final clusters for both stages are shown as heatmaps in Additional File 1: Figure S11 and defined 12 individual chromatin states representing differential combinatorial enrichment of histone modifications, some of which were exclusively found in rings or female gametocytes (Fig. 7A).

#### Chromatin UMAP shows massive chromatin remodelling between parasite stages

To interrogate how global chromatin indexing differs between ring stage parasites and female gametocytes, the bins in the female day 6 gametocyte UMAP were coloured according to their corresponding state in ring stage parasites (Fig. 7B). The resulting UMAP showed approximately the same distribution for the grey coloured bins representing state 1 (none of the studied modifications), meaning that a large proportion of these bins kept their chromatin state between the two life cycle stages. Black state 2 bins, representing H3K9me3 and H2A.Z enrichment in female gametocytes, appeared as state 10 (only H3K9me3) in ring stage parasites, which was also coloured in black. This shows that the majority of loci with state 10 in ring stage parasites (heterochromatic bins defined by H3K9me3 enrichment) acquires state 2 in gametocytes (H3K9me3 and H2A.Z) and is consistent with our observation that H2A.Z is recruited into the heterochromatin compartment in female gametocytes (Fig. 5) and that H3K9me3 coverage is largely similar between rings and gametocytes, except for some specific differentially covered loci (Fig. 4). In contrast to those two areas, the bins carrying other chromatin states were spread across the UMAP reflected in the mixed colours, indicating that these genomic loci were differentially marked between rings and female gametocytes and further demonstrating the global chromatin remodelling we noted before (Fig. 2). For instance, green labelled state 3 bins that are marked by the presence of H3K4me3 and H3R17me2 show dispersion across the UMAP, reflecting the shift of these modifications from intergenic in ring stage parasites to mostly intragenic in gametocytes (Fig. 3). These observations therefore validated our approach.

#### Association of chromatin states with genetic elements

To better understand the epigenetic code in asexual and sexual parasites, we analysed the distribution of the chromatin states in relation to different genetic elements including exons, introns, and intergenic regions (Fig. 7C). To capture differences related to regulatory regions, we further subdivided intergenic regions into tandem, divergent upstream, or convergent downstream regions. In ring stage parasites, intergenic regions containing promoters (divergent or tandem) were mostly defined by state 6 and 11 (H2A.Z with H3K4me3/H3R17me2, with or without H3K27ac), whereas state 1 (none of the modifications) was more dominant in exons and introns. In contrast, in gametocytes state 5 (H2A.Z, H3K27ac, H2A.Zac) and to lesser extent also state 6 dominated promoter containing intergenic regions, while exons and introns were characterized by state 1 or states 3 and 4 (enriched in H3K4me3 and H3R17me2). State 2 or 10 (heterochromatin) were found to occupy exons and intergenic regions in both parasite stages, but were depleted from introns, which is in line with the regulatory function documented for *var* gene introns [37, 87, 88]. Interestingly, a small proportion of promoters was characterized by minor chromatin states such as state 7 (H2A.Z and low levels of H3K27ac and H2A.Zac) or 8 (H2A.Z only) in gametocytes. Together, this demonstrates the high plasticity of the chromatin states between the two parasite stages and suggests different roles of the combinatorial pattern of histone modifications depending on the larger chromatin context.

#### Correlation of promoter chromatin states with *functional gene classes*

After defining the chromatin states present in rings and female gametocytes, we aimed to interrogate the functions and expression profiles of genes carrying specific chromatin states in their upstream regions, ultimately to gain insight into the regulatory roles of the different states. To identify biologically meaningful correlations, we determined which genes carried continuous chromatin state(s) of at least 300 bp in length (representing one to two nucleosomes) within 2 kb upstream of their ATG gene start (Additional File 1: Figure S12). Of note, this allowed for the presence of more than one state per upstream region, which indeed occurred in several genes. The resulting gene lists were subjected to gene ontology (GO) analysis (PlasmoDB, *p*-value < 0.01) (Additional File 5: Table S4) and their expression was plotted across the different parasite stages (Fig. 7D).

Genes featuring state 1 (unmarked by any of the investigated histone modifications) in their promoter in gametocytes or ring stage parasites showed significantly higher expression in gametocytes than in ring stage parasites, which may indicate the presence of an activating mark that was not studied in our set of histone modifications, or lower nucleosomes occupancy, which was previously linked to high transcriptional activity in gametocytes [89, 90]. Genes with state 2 or 10 upstream in female gametocytes and ring stage parasites, respectively, represented mainly variant surface antigens and exported proteins and also included *ap2-g* in ring stage parasites, consistent with heterochromatin silencing of these pathways (Additional File 5: Table S4). States 3 (H3K4me3 and H3R17me2) and 4 (H3K4me3, H3R17me2, H3K27ac low) were detected upstream of only 20 and 69 genes, respectively, in gametocytes and included particularly many nuclear genes involved in “regulation of transcription” (Additional File 5: Table S4). Of note, half of these genes also featured state 3 in rings. In addition, many of the 425 state 3 marked genes in rings were associated with schizont specific functions such as “exit from host cell” and “entry into host”, indicating that H3K4me3/H3R17me2 are required but insufficient for gene activation in the absence of acetylation in rings and poise these genes for activation. State 5 (H2A.Z, H2A.Zac and H3K27ac) and state 6 (H3K4me3, H3R17me2, H3K27ac, H2A.Z and H2A.Zac) were the most prevalent states in female gametocyte and ring stage promoters, respectively, and associated with many functionally diverse genes.

State 7 (220 genes) and state 8 (78 genes) only existed in gametocytes and were predominantly characterized by H2A.Z, either unmodified (state 8) or featuring low levels of acetylation (state 7). Interestingly, these states were also enriched upstream of many genes related to parasite invasion and associated with significantly lower expression levels in female gametocytes than in ring and schizont parasites (Fig. 7D, Additional File 5: Table S4). These states therefore may represent repressive signatures in female gametocytes, possibly in combination with additional modifications associated with suppression of stage-inappropriate genes, as was described for H3K36me3 in immature gametocytes [51]. State 9 (H2A.Z, H3K4me3, H3R17me2) was only identified upstream of 14 genes in gametocytes that mainly belong to multigene families, and thus seems of limited relevance in promoters.

**Fig. 8:**
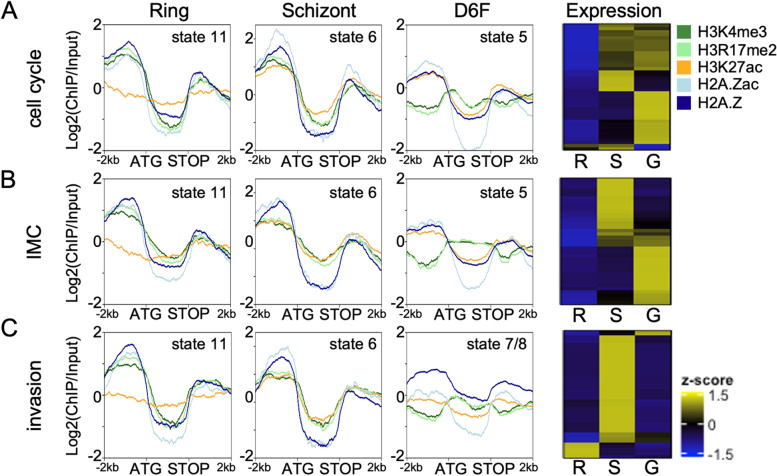
Chromatin states are linked to differential gene expression across parasite stages. The line plots show the log_2_-transformed ChIP/Input coverage of the different histone modifications in the 2 kb upstream region, gene body (ATG to STOP) and 2 kb downstream region in rings, schizonts and day 6 female gametocytes. Heatmaps show the relative expression (FPKM z-scored) of the same groups in rings (R), schizonts (S), and female day 6 gametocytes (G). Gene lists were defined by genes of this group that had a defined chromatin state in rings, resulting in 53 cell cycle genes, 41 IMC genes and 55 invasion genes. **A**) Genes involved in cell cycle. **B**) Genes of the inner membrane complex (IMC). **C**) Genes involved in invasion. The chromatin states observed in each panel are indicated

The ring specific state 11 (H3K4me3, H3R17me2, H2A.Z and H2A.Zac) was prevalent upstream of 778 genes including genes associated with cell cycle, IMC, and parasite invasion. Consistent with their function in later developmental stages, these genes showed significantly lower expression in ring stage parasites compared to schizonts and gametocytes (Fig. 7D). As state 11 is characterized by euchromatic marks with the exception of H3K27ac, this supports the critical function of H3K27ac in stage specific gene expression. To validate this association further and to examine state dynamics over different developmental stages, we included ChIPseq data from schizont stage parasites (H2A.Z, H2A.Zac, H3K4me3, H3R17me2, H3K27ac) and plotted the chromatin profiles in female gametocytes, rings and schizonts for several functional gene groups marked with state 11 in ring stage parasites (Fig. 8). Genes that were annotated under the GO term “cell cycle” showed more accumulation of H3K27ac in their upstream region in schizonts and gametocytes, consistent with state 6 and state 5, respectively, and in line with the temporal expression patterns of these genes (Fig. 8A). The same pattern was observed for genes of the inner membrane complex, which are functionally important and expressed in both schizonts and gametocytes but not in rings (Fig. 8B). In contrast, “invasion genes”, which are highly expressed in schizonts, but neither in rings nor gametocytes carried state 6 in schizonts but state 7 and 8 (low or no histone acetylations) in gametocytes (Fig. 8C). These examples clearly illustrate the dynamics of the different chromatin states and their association with gene expression in the different parasite stages.

Finally, the 47 genes showing state 12 in their promoter in ring stage parasites (characterized by H2A.Z and low levels of H3K27ac), showed a broad range of expression. Interestingly, the most highly expressed genes in this group were associated with protein export and include the major genes coding for the *P. falciparum* translocon, which shuttles proteins across the parasitophorous vacuole membrane into the host cell, including PTEX150, HSP101, Exp2, and the accessory PTEX88, Exp1, and Pf113 [91–96]. Of note, these genes carried state 5 in gametocytes. This exemplifies that certain chromatin states can demarcate and coregulate specific functional gene groups.

In conclusion, we identified chromatin states that are stage specifically enriched in genes linked to particular biological functions and may act as the anchor for recruiting specific chromatin remodelling complexes which fine tune the expression of these genes. In particular we found that H3K27ac occupancy was highly correlated with stage specific gene transcription. In gametocytes, the presence of unacetylated H2A.Z seems to be associated with transcriptional repression.

## Discussion

In this study, we reveal fundamental differences in the transcriptional regulation and epigenetic code of developing male and female gametocytes in comparison to asexual parasites. When analysing genes with peak expression in each stage (Additional File 1: Figure S1), the gene functions for asexual parasites as well as male and female gametocytes were in line with several other proteomic and transcriptomic studies [4, 12, 52, 58, 97–99], however our data set is the first one that differentiates between different maturation states of the gametocyte sexes. Interestingly, we found that male gametocytes were transcriptionally more variable over the course of gametocytogenesis than female gametocytes, consistent with a switch from energy metabolism and proteostasis dominating in immature male gametocytes to DNA replication in preparation for microgametogenesis in mature gametocytes.

We analysed the epigenome of the parasites regarding the histone variant H2A.Z and the histone modifications H2A.Zac, H3K4me3, H3R17me2, H3K27ac and H3K9me3. All modifications were present in gametocytes (Additional File 1: Figure S2), as had been confirmed by mass spectrometry [44]. According to our ChIPseq analyses the coverage of all modifications differed significantly in magnitude and distribution between asexual parasites and gametocytes. The overall lower ChIPseq coverage observed for gametocytes may reflect the previous finding that trophozoites and gametocytes show a more open chromatin structure, and thus lower coverage with histones, due to their higher transcriptional activities [90, 98, 100, 101].

The histone modification H3K4me3 was described to be associated with intergenic regions in asexual parasites, and to accumulate next to the TSS with a strong boundary towards the ATG [34–36, 40, 41], which we could confirm in ring stage parasites. In addition, we found that H3K4me3 globally co-occurs with H3R17me2. This may suggest that the deposition of the two modifications is co-regulated. Histone arginine methylation is mediated by CARM1 in *T. gondii* [71], for which *P. falciparum* encodes a single orthologue PF3D7_0811500. Lysine methylation of H3K4me3 was shown to be catalysed by the methyltransferases SET7 [102] and SET10 [103] in asexual parasites, however, it is unknown whether these proteins are also active in gametocytes or whether other methyltransferases may be responsible. Intriguingly, both histone modifications were globally remodelled between asexual and sexual parasites. In female day 6 gametocytes we could observe broadening of the H3K4me3/H3R17me2 marked areas and a shift into the gene bodies, which are typically depleted of H3K4me3 and covered with H3K4me1 instead in asexual parasites [36]. In contrast to ring stage parasites, in female day 6 gametocytes H3K4me3/H3R17me2 coverage did not correlate with transcription (Fig. 2B), which was also reported for trophozoites, oocysts and sporozoites [74, 98]. A similar repositioning between developmental parasite stages was described for H3K36me2/3, which particularly in stage II gametocytes was enriched in asexual and early gametocyte gene promoters, where it correlated with their repression [51]. In contrast, this modification accumulates in gene bodies in asexual parasites [30].

A recent study investigating histone modification crosstalk by middle-down proteomics revealed that H3K4me3 is strongly linked to H3K23me1 in gametocytes, whereas these two modifications are mutually exclusive in trophozoites [104]. Such an association may mechanistically explain the discrepant enrichment of H3K4me3 and H3R17me2 at distinct genetic elements in gametocytes and asexual stages, for example by differential recruitment of histone methyltransferase complexes to distinct chromatin signatures. So far, H3K23me1 has not been functionally studied in *P. falciparum*.

Interestingly, H3K4me3 and H3R17me2 in female day 6 gametocytes were slightly but significantly elevated in genes that are overrepresented in the ookinete and oocyst stages, suggesting these modifications may poise genes for activation later in the life cycle. The formation of broad H3K4me3 peaks is also well described in mouse oocytes, where they play a role in maternal-to-zygote transition [75, 76, 105]. This phenomenon reverts in the 2-cell embryonic state and was hypothesized to serve the prevention of DNA methylation in the H3K4me3 covered regions during this transition [75, 76, 105]. Such a reversion is consistent with reports that in *Plasmodium* oocysts and sporozoites H3K4me3 is again constrained largely to intergenic regions and start codons [74, 106]. Therefore, the female gametocyte to ookinete development mimics the maternal-to-zygote transition, and the oocyst the 2-cell embryonic state. Broad H3K4me3 coverage reaching from around the TSS into the gene body was also described to occur for genes that are responsive to drought stress in *Arabidopsis thaliana* [107]. Thus, the formation of broad, mostly intragenic H3K4me3/H3R17me2 peaks in female gametocytes could also occur as a stress response, since commitment to gametocytogenesis is triggered by metabolic stress [108] and gametocytes globally rewire their metabolism [6, 109].

The distribution of H3K9me3 in subtelomeric and central clusters was found to be broadly conserved between asexual stages, gametocytes [49, 50, 55] and later mosquito stages [74]. The transcription factor AP2-G is also heterochromatic in asexual parasites but temporarily de-repressed in committed parasites and early gametocytes [15]. Consistent with previous findings, we could show that the gene body of AP2-G but not its promoter region seems to be covered with H3K9me3 in stage II/III gametocytes [81]. Besides differential marking of AP2-G, we observed significant differences between asexual parasites and gametocytes on chromosome 14, in particular for exported, gametocyte specific proteins, which was also described in other studies [49, 50]. Several loci were differentially marked between male and female gametocytes, highlighting the sex specific heterochromatin landscape and pointing to their functional association with males or females, respectively. Interestingly, a putative ncRNA was expressed in female gametocytes and in immature male gametocytes (until stage II/III), but not mature male gametocytes and ring stage parasites, in which the ncRNA locus was covered with H3K9me3. This locus therefore seems to require regulation by heterochromatin and may play a role in sex-specific differentiation. *P. falciparum* sexual commitment is known to be regulated by a lncRNA that supresses the GDV1 activator of the gametocytogenesis transcription factor AP2-G [15]. The function of ncRNAs in regulating sexual differentiation processes is also well documented in other species, for example mating type control of gametogenesis in yeast is regulated by lncRNAs [110].

The histone variants H2A.Z and H2B.Z are typically enriched in euchromatic intergenic regions but depleted from heterochromatic areas in asexual parasites [34–36]. However, specifically in female day 6 gametocytes we observed a striking accumulation of unacetylated H2A.Z/H2B.Z nucleosomes in subtelomeric and central heterochromatin areas, even extending beyond the H3K9me3 delineated boundaries. This phenomenon occurred to a much lesser extent in male gametocytes, in which the H2A.Z patterns were indeed more similar to rings than female gametocytes. Considering the different nuclear biology of male and female gametocytes and the observation that *P. falciparum* gametocytes show an altered nuclear architecture with increased interactions of heterochromatin clusters relative to asexual parasites [49], it will be interesting to investigate in the future whether the variant histones may serve as a scaffold for intra-chromosomal interactions linked to the sex specific reorganisation of the three-dimensional genome structure during gametocytogenesis.

In metazoans it has been described that bivalent domains consisting of the repressive mark H3K27me3 and the euchromatin associated mark H3K4me3 are implicated in silencing of genes needed for embryonic stem cell differentiation [111], and H2A.Z plays a role in the formation and maintenance of these bivalent domains and thereby maintenance of cell identity in embryonic stem cells [32, 112]. Here we also observed co-occurrence of H2A.Z with H3K9me3, albeit not including H3K4me3. Virulence gene expression is reset during transmission of *P. falciparum* [113, 114], thus we speculate that the redistribution of H2A.Z into heterochromatic areas covering multigene families may be linked to resetting these genes for expression in the new host after transmission. The female specific association of H2A.Z may also point to a role in meiotic recombination, for which VSA genes represent prime targets [115]. In the fission yeast *S. pombe* H2A.Z has a function in directing effector proteins that generate double strand breaks at recombination hotspots to initiate meiotic recombination, proposedly by loosening the chromatin structure [116]. Similarly, the knockout of H2A.Z leads to impaired meiotic progression and reduced fitness of the spores in *S. cerevisiae* [117]. This was linked to the association of H2A.Z with the linker of nucleoskeleton and cytoskeleton (LINC)-complex at telomeric ends, which regulates chromosome motion [118].

In *A. thaliana,* the histone variant H2A.W is associated with heterochromatin and promotes heterochromatin condensation dependent on a SPKK motif in its C-terminus [119]. Interestingly, *P. falciparum* H2A.Z carries a similar motif (KPKK) in its C-terminal tail, which suggests it may also fulfil an architectural role in heterochromatin formation and maintenance. The heterochromatin association of H2A.Z could be facilitated by post-translational modifications that would distinguish heterochromatin associated H2A.Z from euchromatin associated H2A.Z, and indeed we observed that H2A.Z was less acetylated in heterochromatic than in euchromatic nucleosomes. In differentiated mouse cells, H2A.Z is not associated with heterochromatin, however its monoubiquitinated form is enriched on the silenced X-chromosome, which is also marked with the repressive mark H3K27me3 [120]. Ubiquitination of H2A.Z has been detected in *P. falciparum* [121], but its functional role and localization in chromatin remains unknown and could mechanistically be linked to heterochromatin targeting of H2A.Z in female gametocytes.

**Fig. 9:**
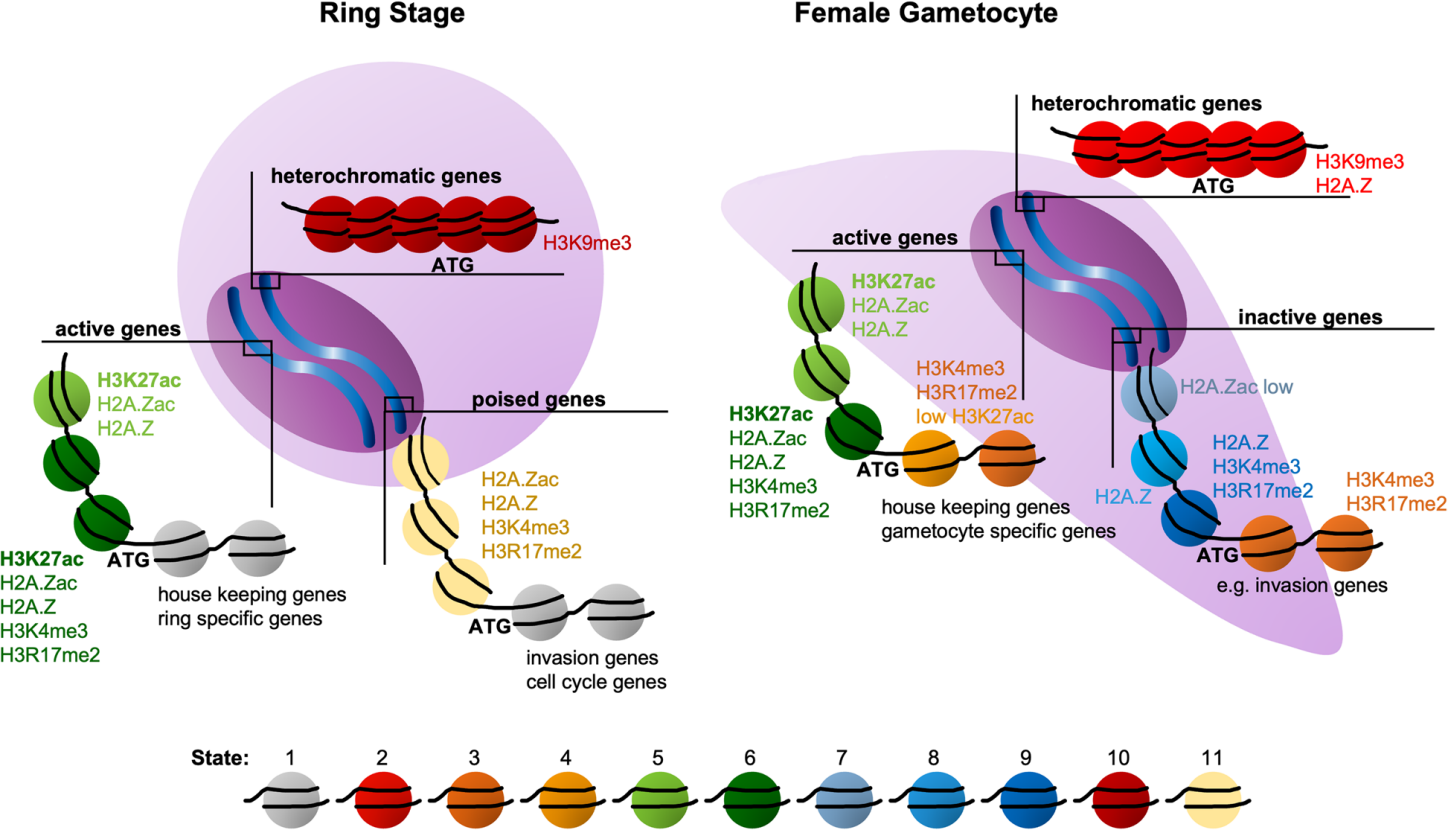
Overview over different chromatin states indexing the genome in ring stage parasites and female gametocytes. A ring stage parasite and a female gametocyte are schematically depicted. The differently coloured spheres represent nucleosomes that carry distinct histone variants and modifications. The ATG indicates the position of the gene starts. Heterochromatic genes are shown as more condensed areas either in dark red as nucleosomes containing only H3K9me3 (state 10 in rings), or in light red as nucleosomes containing both H3K9me3 and H2A.Z (state 2 in female gametocytes). In the euchromatin compartment, regions upstream of active genes contain mostly state 6 nucleosomes (dark green), and to a lesser extent state 5 nucleosomes (light green) in ring stage parasites, whereas this ratio is reversed in female gametocytes with most nucleosomes present in state 5. In female gametocytes, most gene bodies contain state 3 nucleosomes (orange), which are dominated by H3K4me3 and H3R17me2 and some state 4 nucleosomes (light orange), that additionally carry low levels of H3K27ac—in contrast to rings where state 1 (none of the investigated modifications) is predominant (grey). Further, genes poised in ring stage parasites for activation later during the asexual cycle (e.g. invasion, cell cycle) carry state 11 nucleosomes in their upstream region (yellow). In female gametocytes, genes that are inactivated, such as invasion genes, are characterized by state 7, 8, and 9 nucleosomes in their upstream region depicted in shades of blue. State 12 was mostly prevalent in convergent intergenic regions and introns and is therefore not represented in this figure

Of note, acetylated H2A.Z co-occurs with H3K4me3 at active promoters and with H3K27ac at enhancers, while being depleted from bivalent, silent enhancers in embryonic stem cells [32]. In *T. brucei*, H2A.Zac localizes to nucleosomes around the TSS and may be involved in recruiting RNA-Pol II to activate transcription [46]. This is in line with our finding that H2A.Z in the silent heterochromatin compartment in gametocytes was not acetylated. Instead, we could identify H2A.Zac as well as H3K27ac to be mainly located upstream of active genes and to correlate with stage specific gene transcription in female gametocytes and ring stage parasites, but curiously not in male gametocytes. Interestingly, in *P. berghei* H3K9ac correlated with transcription in asexual parasites as well as male gametocytes and ookinetes but not female gametocytes [55]. Conversely, H3K27ac coverage correlated with gene expression in *P. falciparum* sporozoites but not in oocysts [74]. These observations suggest that there may be stage and sex specific preferences in the combinatorial patterns of acetylation marks used for indexing of active promoter regions. To get insight into the interplay of the histone modifications with each other, we defined chromatin states consisting of combinations of the studied histone marks in ring and female gametocytes. Interestingly, there were several chromatin states that were unique to the respective stage and particularly enriched in functionally related genes (summarised in Fig. 9). For example, we found that the invasion genes were mainly marked with state 7 or 8 in gametocytes, which represent transcriptionally repressive states dominated by H2A.Z, whereas subtelomeric regions were marked with H2A.Z and H3K9me3 (state 2). On the basis of our hypothesis that H2A.Z may contribute to the architectural maintenance of the heterochromatin compartment, an interaction between subtelomeric regions and strongly H2A.Z marked, transcriptionally repressed euchromatic regions seems reasonable. This is consistent with the findings of Bunnik et al. 2018, showing that invasion genes interact with subtelomeric regions in gametocytes but not in asexual parasites [49], and aligns with many reports of a dual function of H2A.Z in transcriptional activation and repression [31]. Ultimately, we found that in female gametocytes and ring stages, H3K27ac was the best predictor of gene activity of all modifications studied here, suggesting this mark is a key signal for directing RNA Polymerase activity to stage specifically expressed genes. Thus, it will be very relevant to identify the reader proteins that can interpret this code and direct stage specific gene expression.

## Conclusions

In conclusion, our data demonstrate the complexity of the histone code in genome organization of the malaria parasite *P. falciparum* and reveal fundamental differences not only between sexual and asexual parasites, but in particular also between male and female gametocytes. Clearly, chromatin modifications regulating transcription in gametocytes extend beyond the functionally known combinatorial patterns of histone modifications, and we need to take other, recently identified posttranslational modifications into consideration to better understand the processes regulating sexual differentiation. Asexual and sexual parasites differ significantly in their cellular metabolism, and recent publications increasingly demonstrate how changes in metabolites can govern differential gene expression programs by causing alternative chromatin modifications [122]. Several recent publications have used proteomics approaches to gain insight into differential histone modifications prevalent in developmental stages of the malaria life cycle. Our dataset provides an important source for future studies further investigating epigenetic regulation of differentiation processes in *P. falciparum*.

## Methods

### Parasite lines

To isolate male and female gametocytes, the previously described 3D7::ABCG2-GFP parasite line was used [56]. For detection of histone modifications in asexual and sexual parasites, NF54 (patient isolate E) parasites were used.

### *Plasmodium falciparum* parasite culture

Parasites were cultivated as described previously [123], at 5% haematocrit in complete culture medium containing RPMI-1640 (Gibco, Thermo Fisher Scientific #21,875–091) complemented with 25 mM HEPES pH 7.3 and 20 µg/ml gentamicin (Thermo Fisher Scientific) and supplemented with 5% AlbuMAX stock solution pH 7.3 (25 g AlbuMAX II Lipid-Rich BSA (Thermo Fisher Scientific), 500 ml RPMI-1640, 2.97 g HEPES, 200 µl gentamycin (50 mg/ml), 0.67 g Hypoxanthine) and 5% human AB Rh + serum purchased from the Bavarian Red Cross Service (BRK). The parasites were propagated in erythrocytes of blood group 0 + or A + (purchased from BRK) in a gas environment of 1% O_2_, 5% CO_2_, and 94% N_2_ at 37 °C.

### Gametocyte culture

To induce gametocytogenesis, a tightly synchronised ring stage culture at about 6–8% parasitemia was stressed by adding 50% fresh medium to the partially spent medium, thereby decreasing the haematocrit (day -2) [108]. At about 38 hpi, the trophozoites were split to 2% parasitemia and the medium was replaced with fresh medium (day -1). The next day (day 0), the medium was removed and the committed ring stage parasites were cultivated in fresh medium containing 50 mM N-Acetyl-D-Glucosamine (Sigma Aldrich) until day 6. Medium was exchanged every second day. Mature stage V gametocytes were observed around day 10.

### Western blot analysis

For the preparation of complete cell lysates, the erythrocytes were lysed on ice by resuspension in 0.075% Saponin (Sigma Aldrich) in PBS. Upon lysis, the erythrocytes were centrifuged at 2300 × g for 10 min at 4 °C. The pellet was washed twice with PBS, resuspended with 2 × Laemmli-Buffer (10^6^ parasites per µl), heated for 5 min at 95 °C and subsequently spun at 14,000 × g for 10 min. Finally, the supernatant was loaded on a 4 – 12% Bis–Tris gel (Invitrogen) in NuPAGE MES SDS running buffer (Thermo Fisher Scientific) and run for 1.25 h at 120 V. Thereafter, proteins were transferred onto Nitrocellulose membranes (GE Healthcare). The blotting was conducted at 400 mA for 1 h in transfer buffer (0.198 M glycine, 20% methanol, 1% SDS, 0.25 mM Tris) in a Mini-PROTEAN Tetra Vertical Electrophoresis Cell (Biorad) blotting chamber. After blotting, the membranes were blocked for 2 h in blocking solution (5% non-fat milk powder in TBS with 0.05% Tween). The first antibody was applied in blocking solution with 0.02% sodium azide at 4 °C overnight. After washing the membrane in TBS-T, horse radish peroxidase (HRP) conjugated secondary antibodies in blocking solution were applied to the membrane for 2 h at RT. Detection was done using chemiluminescent HRP substrate (Immobilon ® Western Millipore) and imaging with a ChemiLux Imager (Intas Science Imaging).

### Immunofluorescence analysis

Parasites were smeared onto glass slides and fixed in 10% Methanol / 90% Aceton, air dried and stored at -20 °C. The slide was rehydrated in PBS for 10 min and subsequently blocked in 1% BSA in PBS for 30 min. The GFP-antibody (Additional File 6: Table S5) was then applied for 2 h in blocking solution. After washing the slides 3 times for 5 min in PBS, the slide was incubated with the secondary anti-rabbit Alexa-488 antibody and Hoechst 33,342 (Additional File 6: Table S5) for 2 h in the dark. Finally, unbound antibody was washed off again 3 times for 5 min in PBS and the slides were mounted over night with Mowiol (Merck Millipore) including DABCO (Diazabicyclooctan Sigma Aldrich). The slides were imaged using a Zeiss Axio Observer and analysed with Zeiss ZEN Pro software 3.0.

### Flow cytometric separation of male and female gametocytes

Flow cytometry was used to sort 3D7::ABCG2-GFP gametocytes according to their viability and sex. For sorting, the parasites were purified from the total culture using MACS CS columns (MACS Miltenyi) at least two days before sorting and then further cultivated in complete culture medium. As outlined in Additional File 2: Table S1, in some preparations the gametocytes were stained with MitoTracker™ Deep Red (Invitrogen) right before FACS sorting. For this, the MACS enriched gametocytes were spun down and resuspended with RPMI-1640 to a cell density of 5 × 10^7^ cells per ml. MitoTracker™ Deep Red 1 mM working solution was then added 1:10,000 and incubated for 2 min at 37 °C in the dark. The cells were spun down and washed once in warm RPMI-1640 and finally resuspended in PBS for sorting either at the University of Melbourne Cytometry Platform on a BD FACS Aria III or at the FACS Core Facility University Erlangen on a MoFlo Astrios EQ 1 at 37 °C. Parasites were sorted into two populations: For three samples, parasites were sorted into a GFP positive female gametocyte population and a GFP low male population and for further three samples the parasites were sorted into MitoTracker™ and GFP double positive parasites as viable female gametocytes, and MitoTracker™ positive and GFP low parasites as viable male gametocytes (Additional File 2: Table S1). The sorts and subsequent harvest of the cells were conducted on day 4, 6 and 10 of gametocytogenesis for RNA and on day 6 of gametocytogenesis for chromatin.

### RNA isolation

For RNA extraction, FACS sorted gametocytes or culture pellets of ring and schizont stage infected erythrocytes were resuspended in 10 times the pellet volume TRIzol® Reagent (Life Technologies). RNA was extracted by addition of 0.2 volumes chloroform followed by 30 min centrifugation at full speed and 4 °C. The aqueous layer containing the RNA was purified using the RNeasy Mini Kit (QIAGEN 74,104). DNA was removed by on-column digest and an additional in solution digest with DNase I (QIAGEN 79,254). The concentration of the resulting RNA was measured by NanoDrop 1000 Spectrophotometer (Thermo Fisher Scientific) and the purity was determined by qPCR with primers specific for SBP1.

### Chromatin immunoprecipitation

Sorbitol synchronized cultures of ring stage parasites (4–14 hpi), schizont stage parasites (38–48 hpi) or purified gametocytes were crosslinked at a final concentration of 1% PFA (16% PFA Thermo Fisher Scientific) for 10 min at 37 °C. The reaction was quenched for 5 min on ice with 125 mM glycine. The infected erythrocytes were then pelleted (5 min at 800 × g at 4 °C) and permeabilized in 0.075% saponin in PBS on ice to release haemoglobin. The parasite pellets were then washed twice with PBS (2300 × g at 4 °C for 5 min) and resuspended in 2 ml cold lysis buffer (10 mM Hepes pH 7.9, 10 mM KCl, 0.1 mM EDTA pH 8.0, 0.1 mM EGTA pH 8.0, 1 mM DTT, 1 × Protease Inhibitor (Roche)) per 10^8^ parasites. The mix was transferred to a prechilled 2 ml tissue grinder (Kimble) and incubated on ice for 30 min. Subsequently, IGEPAL CA-630 was added to a final concentration of 0.25% and the parasites were lysed with 100 strokes. The lysed parasites were then centrifuged for 10 min at 20,000 × g, 4 °C to pellet the nuclei.

The nuclei were then lysed by addition of 200 µl SDS Lysis Buffer (1% SDS, 10 mM EDTA, 50 mM Tris pH 8.1, 1 × Protease Inhibitor) per 10^8^ nuclei and sonication for 2 times 8 cycles of 30 s ON and OFF in a chilled Bioruptor® Sonication System (Diagenode). The sheared material was spun (12,500 × g for 10 min at 4 °C) and the supernatant was then transferred and diluted 1:10 with ChIP Dilution Buffer (0.01% SDS, 1.1% Triton X-100, 1.2 mM EDTA, 16.7 mM Tris–HCl pH 8.1, 150 mM NaCl). The diluted chromatin was then pre-cleared with 30 µl Protein G Sepharose 4 Fast Flow (GE Healthcare) for 1 h at 4 °C under agitation. The beads were prepared for the immunoprecipitation (IP) by blocking in 0.1% BSA (Sigma Aldrich) in ChIP Dilution Buffer for 10 min. After two washing steps with ChIP Dilution Buffer, the beads were preincubated with the respective antibodies (Additional File 6: Table S5) for the IP. Per IP, 15 µl agarose G beads were used. After preclearing of the chromatin and prebinding of the antibody to the beads, 50 µl chromatin were taken as Input fraction, and the beads were resuspended with chromatin and incubated overnight under agitation at 4 °C. Subsequently the beads were washed with 1 ml washing buffer each: first at 4 °C with Low Salt Wash Buffer (0.1% SDS, 1% Triton X-100, 2 mM EDTA, 20 mM Tris–HCl pH 8.1, 150 mM NaCl), then High Salt Wash Buffer (0.1% SDS, 1% Triton X-100, 2 mM EDTA, 20 mM Tris–HCl pH 8.1, 500 mM NaCl), then LiCl Wash Buffer (0.25 M LiCl, 1% Igepal, 1% Sodium-Deoxycholate, 1 mM EDTA, 10 mM Tris–HCl pH 8.1), then twice at RT with TE (10 mM Tris–HCl pH 8, 1 mM EDTA pH 8). The immunoprecipitated chromatin was eluted from the beads with 200 µl Elution Buffer (2% SDS, 200 mM NaHCO3) for 15 min at RT. The Input sample was also diluted to the same volume with Elution Buffer. 500 mM NaCl was added for decrosslinking (45 °C over night). Finally, the proteins were digested by addition of 2 µl Proteinase K (Thermo Fisher Scientific) for 1 h at 37 °C, and DNA was purified using the MinElute Kit (QIAGEN 28,006).

### Library preparation and Illumina sequencing

Libraries were prepared from the ChIP and RNA samples. For ChIP sequencing the Accel-NGS™ 2S Plus DNA Library Kit for Illumina platforms (SWI Swift Bioscience) was used according to the manufacturer’s guidelines, including the Accel-NGS™ 2S Indexing Kit (SWI Swift Bioscience) until the PCR amplification step. The RNA sequencing libraries were prepared using the NEBNext Ultra II directional RNA Library Prep Kit for Illumina (NEB) with the NEBNext Poly(A) mRNA Magnetic Isolation module (NEB) and the NEBNext Multiplex Oligos for Illumina (NEB). For both library types, Ampure XP beads (Beckman Coulter) were used for the purification steps and the library amplification step was performed with 12–15 cycles using KAPA Polymerase (KAPA HiFi PCR Kit Roche. The PCR program used for the library amplification consisted of an initial denaturation step at 98 °C, 15 cycles of 15 s 98 °C and 2 min 65 °C, then 5 min 65 °C and finally cooling down to 4 °C.

Quality control for the libraries was done using Qubit (Thermo Fisher Scientific) with the dsDNA HS Assay Kit (Thermo Fisher Scientific Q32854) or the RNA HS Assay Kit (Thermo Fisher Scientific Q32855) to determine the concentration, and subsequently on a TapeStation (Agilent Technologies) using the D5000 kit according to the manufacturers protocol to determine molarity from the average fragment size. The analysis was done using the TapeStation Analysis Software 3.1.1., with a size range of 150 bp to 2000 bp. The libraries were subsequently pooled to a final concentration of 5 nM and sequenced as 100–150 bp paired end reads for RNAseq and 100–150 bp paired end reads for ChIP-Seq on an Illumina HiSeq 2500 instrument (Additional File 2: Table S1). Schizont ChIP samples were sequenced as 150 bp paired end reads on a Illumina HiSeq 4000 instrument.

### Data analysis

The raw data was processed on the Spartan HPC (University of Melbourne). ChIPseq data in fastq format was first quality checked using FastQC 0.11.8 (https://www.bioinformatics.babraham.ac.uk/projects/fastqc/). The files were then trimmed to remove remaining adaptors and low quality reads using TrimGalore 0.6.4 (https://github.com/FelixKrueger/TrimGalore). If necessary, the files were further trimmed using Cutadapt 2.8 [124] specifying the overrepresented sequences. Subsequently the reads were mapped to the reference genome of *Plasmodium falciparum* (PlasmoDB 3D7 version 28) using Bowtie2 version 2.3.4.3 with default settings for paired-end reads [125]. The resulting sam files were then transformed into bam files by samtools 1.9 and bam files of biological replicates were merged [126]. Correlation between the replicates was checked using deeptools 3.1.2 multiBamSummary and correlation dot plots were generated using multiBigwigSummary [127]. The individual and merged bam files were visualised by transforming them into bigwig files using deeptools 3.1.2 bamCoverage and bamCompare. Visualisation of bigwig files was done using IGV 2.6.3 [128] and pyGenometracks 3.6 [129] using Python 3.8.5. The peaks for the histone modifications were called using MACS2 2.1.2.1 [73] compared by searching their closest genomic feature (gene or ATG) using bedtools 2.28.0 closest or intersect [130]. General differences between the coverage of samples were investigated using CSAW 1.22.1 [72]. Bedtools 2.28.0 multicov was used to generate an input file of H3K9me3 coverage 500 bp around the ATG of every gene to predict the probability of it being heterochromatic using a bivariate gaussian distribution by mixtools 1.2.0 NormalmixEM [131] in R-Studio 1.3.1093 [132]. If the probability for being heterochromatic was > 0.99999, the gene was assumed to be heterochromatic. Heatmaps were generated using deeptools 3.3.0 computeMatrix and subsequent plotHeatmap. Violin plots and pie charts were generated using ggplot2 3.3.2 [133]. Uniform Manifold Approximation and Projection for Dimension Reduction figures were generated by transforming bigwig files with bin size 50 into bedgraph files, merging them into a general data matrix and using scanpy version 1.8.1 [134] for clustering and plotting. The clusters were initially generated using the leiden algorithm [135], then fused according to their similarity. Ambiguous clusters were divided using additional k-means clustering. The final clusters were defined as different chromatin states. States found for each bin were added as observation to the data table, allowing to colour the ring UMAP by chromatin state in gametocytes and to calculate percentages of bins with conserved states. Chromatin states in upstream regions of genes were determined using bedtools closest, states intersecting different genomic elements were determined using bedtools intersect [130]. Percentages of bins with the same state were calculated from the data matrix. Gene ontology (GO) analysis was done using PlasmoDB Release 53, *p*-value < 0.01. The list of ookinete specific genes was retrieved from PlasmoDB using the following settings: > fivefold overexpressed in ookinetes compared to all other stages on a floor of 100 reads [48].

The RNAseq data was also processed on Spartan HPC and quality checked using FastQC 0.11.8. The trimming and alignment was done using STAR 2.7.3 [136]. Subsequently, the files were transformed into bamfiles using samtools 1.9 and the correlation of replicates was checked using deeptools 3.1.2 multiBamSummary [127] before merging the replicate files. The FPKM (fragments per kilobase million) value was calculated using Cufflinks 2.2.1 [137] and differential gene expression was analysed using Cuffdiff between the merged replicates [138]. To divide sense and antisense transcripts, sam flags were used and the transcripts were intersected with annotated genes according to their strand information using bedtools. Lists of stage specific genes were generated from sense transcripts by k-means clustering of z-scored data, resulting in lists of genes with peak expression in the respective stages. Heatmaps were generated using ComplexHeatmap 2.4.3 [139] in R-Studio 1.3.1093. GO analysis was done using PlasmoDB Release 53 and subsequently Revigo (tiny 0.4; Resnik (normalised) as semantic similarity measure) [140].

## Supplementary Information

**Additional file 1: Figure S1.** Validation of male and female gametocyte populations. A and B) Parasites expressing GFP-tagged ABCG2 [56] were tightly synchronised and induced to generate gametocytes. On day 4 of gametocytogenesis, parasites were stained with MitoTracker^TM^ Deep Red and FACS-sorted according to their GFP signal. The mitotracker/GFP double positive (G+M+) female gametocyte population and the mitotracker positive, GFP negative (G-M+) male gametocyte population were then cultivated and reanalysed after two days by A) live microscopy and B) FACS. The GFP fluorescence intensity of the mitotracker positive cells was determined and significant differences between the populations determined by t-test. C) Heatmap showing the z-score transformed sense expression of several previously described stage specific marker genes (colour coded) determined by RNAseq to validate the cellular identity of the harvested parasite populations. D4 = Day 4, D6 = Day 6, D10 = Day 10, M = male gametocyte, F = female gametocyte. Male gametocytes: day 4: *n*=3, day 6: *n*=2, day 10: *n*=1; female gametocytes: day 4: *n*=2, day 6: *n*=4, day 10: *n*=4; ring stage parasites: *n*=2, schizont stage parasites: *n*=1. E) Heatmap of the spearman correlation coefficients between the RNAseq data sets from the different stages. Male gametocytes: day 4: *n*=3, day 6: *n*=2, day 10: *n*=1; female gametocytes: day 4: *n*=2, day 6: *n*=4, day 10: *n*=4; ring stage parasites: *n*=2, schizont stage parasites: *n*=1. **Figure S2.** Western blot of histone variants and modifications in bulk gametocyte cultures. H3 served as a loading control and Pfs16 as gametocyte specific marker. HP1 = heterochromatin protein 1.**Figure S3.** Overview over log_2_-transformed ChIP/Input coverage across all 14 *P. falciparum* chromosomes in female gametocytes and ring stage parasites. H3K4me3 (*n*=2 for female gametocytes and ring stage parasites), H3R17me2 (*n*=2 for female gametocytes and ring stage parasites), H3K27ac (*n*=3 for female gametocytes, *n*=2 for ring stage parasites), H2A.Z (*n*=6 for female gametocytes, *n*=2 for ring stage parasites)*,* H3K9me3 (*n*=5 for female gametocytes*, n*=2 for ring stage parasites). **Figure S4.** Heatmaps showing the log_2_-transformed ChIP/Input coverage of all genes and their 2 kb up/downstream region for each modification sorted into groups according to their expression. The black bar in the silent genes represents silent mitochondrial and apicoplast genes, that carry no histone modifications. A) Day 6 female gametocytes. B) Day 6 male gametocytes. C) Ring stage parasites. H3K4me3 (*n*=2 for female gametocytes and ring stage parasites), H3R17me2 (*n*=2 for female gametocytes and ring stage parasites), H3K27ac (*n*=3 for female gametocytes, *n*=2 for ring stage parasites), H2A.Z (*n*=6 for female gametocytes, *n*=2 for male gametocytes, *n*=2 for ring stage parasites), H2A.Zac (*n*=2 for female gametocytes, *n*=1 for male gametocytes), H3K9me3 (*n*=5 for female gametocytes, *n*=2 for male gametocytes, *n*=2 for ring stage parasites). **Figure S5.** Global ChIPseq and RNAseq profiles for regions of particular interest in female gametocytes and ring stage parasites. Genes are marked depending on their genomic orientation in either green (+ genes) or orange (- genes). Introns are shaded in light green or dark red. The position of the ATG is marked by a dashed line. Transcripts (FPKM) are shown in black. Regions shown: SBP1 from 65.5 kbp to 72 kbp, AP2-G from 904.7 kbp to 918 kbp, Pfs230 from 368.4 kbp to 381.2 kbp, PUF1 from 773.2 kbp to 783.5 kbp. H3K4me3 (*n*=2 for female gametocytes and ring stage parasites), H3R17me2 (*n*=2 for female gametocytes and ring stage parasites), H3K27ac (*n*=3 for female gametocytes, *n*=2 for ring stage parasites), H2A.Z (*n*=6 for female gametocytes, *n*=2 for ring stage parasites)*,* H3K9me3 (*n*=5 for female gametocytes*, n*=2 for ring stage parasites). **Figure S6.** H3K4me3 and H3R17me2 coverage in genes with peak expression in asexual or sexual parasites. Average log2-transformed ChIP/Input coverage plots from two biological replicates for H3K4me3 and H3R17me2 in day 6 female gametocytes and ring stage parasites over genes with peak expression in early (cluster 3) and late (cluster 4) female gametocytes and rings (cluster 1). **Figure S7.** H3K4me3/H3R17me2 coverage in gametocytes is significantly increased in ookinete and oocyst specific genes. Log_2_-transformed ChIP/Input coverage plots from two biological replicates for H3K4me3 and H3R17me2 in day 6 female gametocytes and ring stage parasites over ookinete genes upregulated after JIB-04 treatment ([18], available as Supplementary Table), ookinete genes ([48], available on PlasmoDB) and oocyst genes ([74], available on PlasmoDB) compared to all genes. Plotted is the cumulative coverage over the indicated regions for each gene group. Significant differences between the average coverage of all genes and the specific gene groups were determined by one-way ANOVA and are indicated with stars over the bars, * *p* < 0.05, ** *p* < 0.01, *** *p* < 0.001. **Figure S8.** Heterochromatin and transcription profile of GECO (gametocyte erythrocyte cytosolic protein), region shown from 2167 kbp to 2170 kbp. RNAseq data: Male gametocytes: day 4: *n*=3, day 6: *n*=2, day 10: *n*=1; female gametocytes: day 4: *n*=2, day 6: *n*=4, day 10: n=4; ring stage parasites: *n*=2; ChIPseq data: H3K9me3 (*n*=5 for female gametocytes*, n*=2 for male gametocytes, *n*=2 for ring stage parasites). **Figure S9.** H2A.Z is associated with heterochromatic areas in day 6 female gametocytes. Overview over chromosome 7 showing log_2_-transformed ChIP/Input coverage for H2A.Z (*n*=6 for female gametocytes, *n*=2 for male gametocytes, *n*=2 for ring stage parasites) and H3K9me3 (*n*=5 for female gametocytes, *n*=2 for male gametocytes, *n*=2 for ring stage parasites) in all stages, H2A.Zac in male (*n*=1) and female gametocytes (*n*=2), and H2B.Z in female gametocytes (*n*=1). Zoom into internal heterochromatic cluster on chromosome 7. Genes are marked in either green (+ genes) or orange (- genes). Introns are marked either in light green or dark red, depending on gene orientation. **Figure S10.** H3K27ac and H2A.Zac coverage is unchanged in translationally repressed genes. Average log2-transformed ChIP/Input coverage plots for H2A.Zac (*n*=2 for female gametocytes, *n*=1 for male gametocytes) in day 6 male and female gametocytes and H3K27ac in female gametocytes (*n*=3) over all genes in comparison to genes ranking in the top expressed tercile (excluding DOZI-repressed genes) and DOZI-repressed genes in day 6 female gametocytes. **Figure S11.** Heat maps of chromatin states discovered in female day 6 gametocyte and ring stage parasites. Heatmap showing the log_2_-transformed ChIP/Input coverage of the studied histone modifications over all 50 bp bins represented in each of the A) female day 6 gametocyte states and B) ring stage parasite states. **Figure S12.** Length distribution of chromatin states. Neighbouring bins with identical chromatin states were fused and the lengths of the continuous regions with the same chromatin state were plotted as violin plots for female day 6 gametocytes and ring stage parasites.

**Additional file 2:** **Table S1.** Overview over RNAseq and ChIPseq replicates generated in this study.

**Additional file 3:** **Table S2.** RNAseq gene expression data (FPKM values) and gene clusters including GO analyses.

**Additional file 4:** **Table S3.** Comparison of heterochromatic loci with published data from Bunnik et al. 2018 [49] and Fraschka et al. 2018 [50].

**Additional file 5:** **Table S4.** List of genes associated with different chromatin states upstream in female gametocytes and ring stage parasites including Gene Ontology analyses.

**Additional file 6:** **Table S5.** Antibodies used for western blot (WB), immunofluorescence analysis (IFA), and chromatin immunoprecipitation (ChIP).

**Additional file 7.** Uncropped western blots shown in Figure S2.

## Data Availability

All data generated or analysed during this study are included in this published article, its supplementary information files and publicly available repositories. RNAseq data generated for this study are available under GEO accession number GSE180985 (https://www.ncbi.nlm.nih.gov/geo/query/acc.cgi?acc=GSE180985) [141]. ChIPseq data generated for this study are available under GEO accession number GSE202214 (https://www.ncbi.nlm.nih.gov/geo/query/acc.cgi?acc=GSE202214) [142]. In addition, publicly available datasets from [48] (https://plasmodb.org/plasmo/app/record/dataset/DS_66f9e70b8a), and [74] (https://plasmodb.org/plasmo/app/record/dataset/DS_2aa311d6a0) were obtained from PlasmoDB. Datasets for deregulated genes upon drug treatment of parasites were directly obtained from supplementary data of the respective publications [21] (https://www.frontiersin.org/articles/file/downloadfile/259530_supplementary-materials_tables_2_xlsx/octet-stream/Table%202.XLSX/1/259530) and [18] (https://pubs.acs.org/doi/suppl/10.1021/acsinfecdis.9b00455/suppl_file/id9b00455_si_002.xlsx).

## Abbreviations

ABCG2: ABC transporter G family member 2
ac: Acetylation
ACS1a: Acyl-CoA synthetase 1a
AdoMetDC/ODC: S-adenosylmethionine decarboxylase/ornithine decarboxylase
bp: Base pairs
CARM1: Coactivator associated arginine methyltransferase 1
ChIPseq: Chromatin Immunoprecipitation Sequencing
Clag3: Cytoadherence linked asexual protein 3
CSAW: ChIP-Seq Analysis with Windows
DOZI: Development of zygotes inhibited
FACS: Fluorescence Activated Cell Sorting
FPKM: Fragments per kilobase million
GDV1: Gametocyte development protein 1
GECO: Gametocyte erythrocyte cytosolic protein
gexp17: Early gametocyte exported protein 17
GFP: Green fluorescent protein
GO: Gene ontology
H2A.Z: Histone variant H2A.Z
H2B.Z: Histone variant H2B.Z
H3: Histone H3
HP1: Heterochromatin protein 1
HSP: Heat shock protein
IMC: Inner membrane complex
K: Lysine
Kb: Kilobase
lncRNA: Long noncoding RNA
me: Methylated
ncRNA: Noncoding RNA
PCA: Principal component analysis
*P. falciparum*: *Plasmodium falciparum*
PfEMP1: *Plasmodium falciparum* Erythrocyte membrane protein 1
Pfs230: *Plasmodium falciparum* 6-Cysteine protein P230
PHIST: *Plasmodium* Helical Interspersed Subtelomeric
PTEX: *Plasmodium* Translocon of exported proteins
PUF1: Pumilio RNA binding family 1
RNA: Ribonucleic acid
RNAseq: RNA Sequencing
SET: (Su[var]3–9, Enhancer of Zeste, Trithorax)-domain-containing
TCA: Tricarboxylic acid
TSA: Trichostatin A
TSS: Transcription start site
UMAP: Uniform Manifold Approximation and Projection
VSA: Variant surface antigen
