# Supplementary material for: Plasmodium falciparum gametocytes display global chromatin remodelling during sexual differentiation
Source: BMC Biol. 2023 Apr 3;21:65. doi: 10.1186/s12915-023-01568-4 (PMC10071754; doi:10.1186/s12915-023-01568-4)
Supplement: Supplementary file 1 — Additional file 1: Figure S1. Validation of male and female gametocyte populations. A and B) Parasites expressing GFP-tagged ABCG2 [56] were tightly synchronised and induced to generate gametocytes. On day 4 of gametocytogenesis, parasites were stained with MitoTrackerTM Deep Red and FACS-sorted according to their GFP signal. The mitotracker/GFP double positive (G+M+) female gametocyte population and the mitotracker positive, GFP negative (G-M+) male gametocyte population were then cultivated and reanalysed after two days by A) live microscopy and B) FACS. The GFP fluorescence intensity of the mitotracker positive cells was determined and significant differences between the populations determined by t-test. C) Heatmap showing the z-score transformed sense expression of several previously described stage specific marker genes (colour coded) determined by RNAseq to validate the cellular identity of the harvested parasite populations. D4 = Day 4, D6 = Day 6, D10 = Day 10, M = male gametocyte, F = female gametocyte. Male gametocytes: day 4: n=3, day 6: n=2, day 10: n=1; female gametocytes: day 4: n=2, day 6: n=4, day 10: n=4; ring stage parasites: n=2, schizont stage parasites: n=1. E) Heatmap of the spearman correlation coefficients between the RNAseq data sets from the different stages. Male gametocytes: day 4: n=3, day 6: n=2, day 10: n=1; female gametocytes: day 4: n=2, day 6: n=4, day 10: n=4; ring stage parasites: n=2, schizont stage parasites: n=1. Figure S2. Western blot of histone variants and modifications in bulk gametocyte cultures. H3 served as a loading control and Pfs16 as gametocyte specific marker. HP1 = heterochromatin protein 1.Figure S3. Overview over log2-transformed ChIP/Input coverage across all 14 P. falciparum chromosomes in female gametocytes and ring stage parasites. H3K4me3 (n=2 for female gametocytes and ring stage parasites), H3R17me2 (n=2 for female gametocytes and ring stage parasites), H3K27ac (n=3 for female gametocytes, n=2 for ring st [file 12915_2023_1568_MOESM1_ESM.pdf]

Additional File 1

Supplementary Figures S1 –S12

Plasmodium falciparum gametocytes display global chromatin remodelling during sexual differentiation

Myriam D. Jeninga, Jingyi Tang, Shamista A. Selvarajah, Alexander G. Maier,

Michael F. Duffy, Michaela Petter

Figure S1

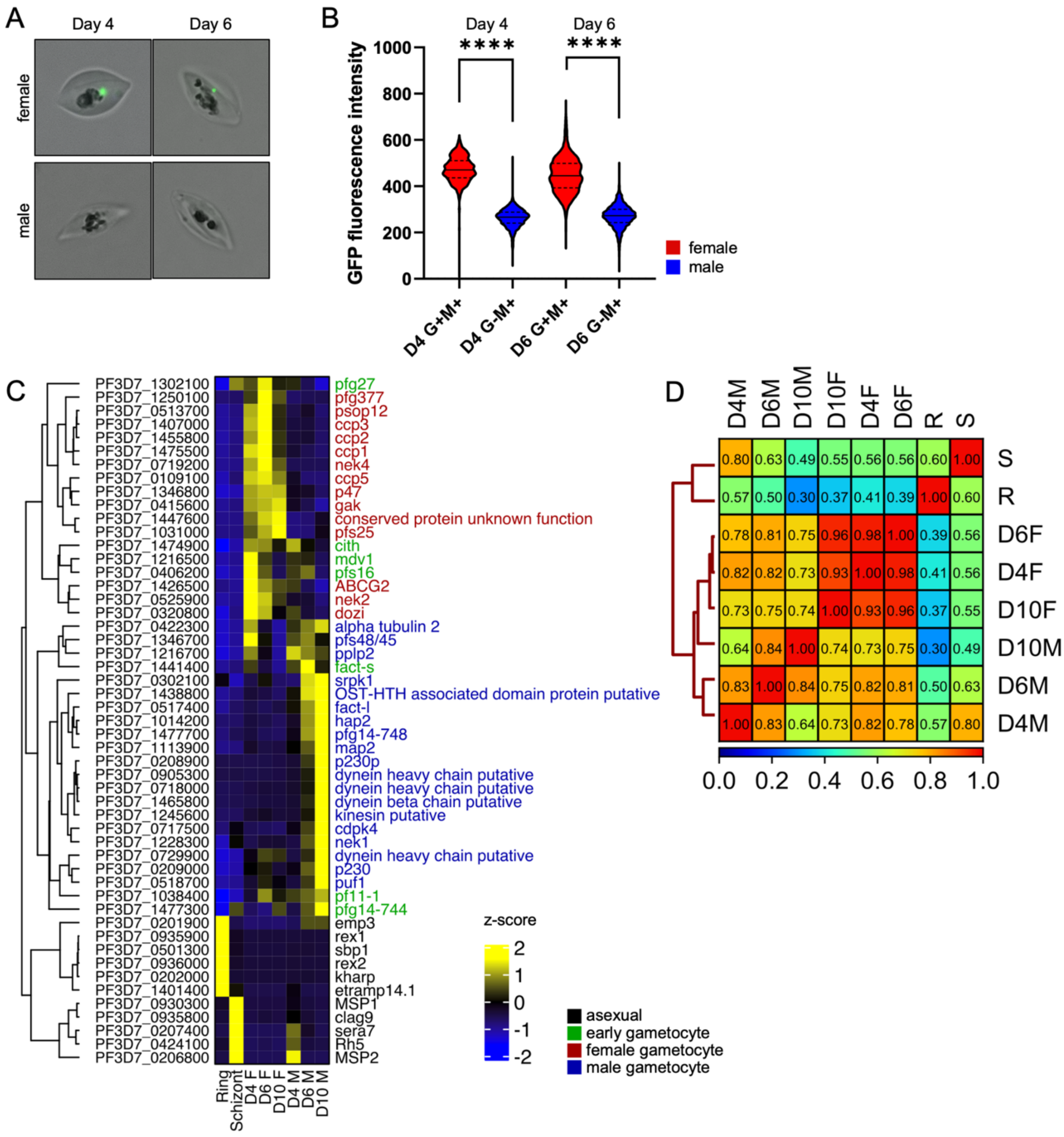

Figure S1: Validation of male and female gametocyte populations. A and B) Parasites expressing GFP-tagged ABCG2 [56] were tightly synchronised and induced to generate gametocytes. On day 4 of gametocytogenesis, parasites were stained with MitoTrackerTM Deep Red and FACS-sorted according to their GFP signal. The mitotracker/GFP double positive (G+M+) female gametocyte population and the mitotracker positive, GFP negative (G-M+) male gametocyte population were then cultivated and reanalysed after two days by A) live microscopy and B) FACS. The GFP fluorescence intensity of the mitotracker positive cells was determined and significant differences between the populations determined by t-test. C) Heatmap showing the z-score transformed sense expression of several previously described stage specific marker genes (colour coded) determined by RNAseq to

validate the cellular identity of the harvested parasite populations. D4 = Day 4, D6 = Day 6, D10 = Day 10, M = male gametocyte, F = female gametocyte. Male gametocytes: day 4: n=3, day 6: n=2, day 10: n=1; female gametocytes: day 4: n=2, day 6: n=4, day 10: n=4; ring stage parasites: n=2, schizont stage parasites: n=1. E) Heatmap of the spearman correlation coefficients between the RNAseq data sets from the different stages. Male gametocytes: day 4: n=3, day 6: n=2, day 10: n=1; female gametocytes: day 4: n=2, day 6: n=4, day 10: n=4; ring stage parasites: n=2, schizont stage parasites: n=1.

Figure S2

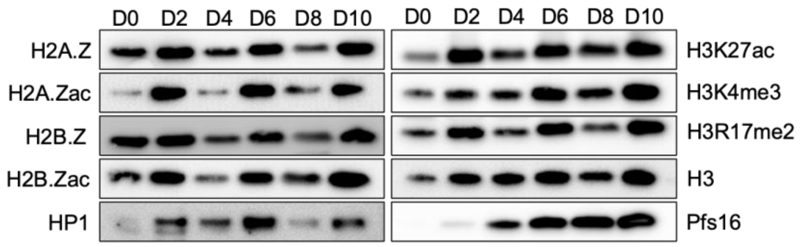

Figure S2: Western blot of histone variants and modifications in bulk gametocyte cultures. H3 served as a loading control and Pfs16 as gametocyte specific marker. HP1 = heterochromatin protein 1.

Figure S3

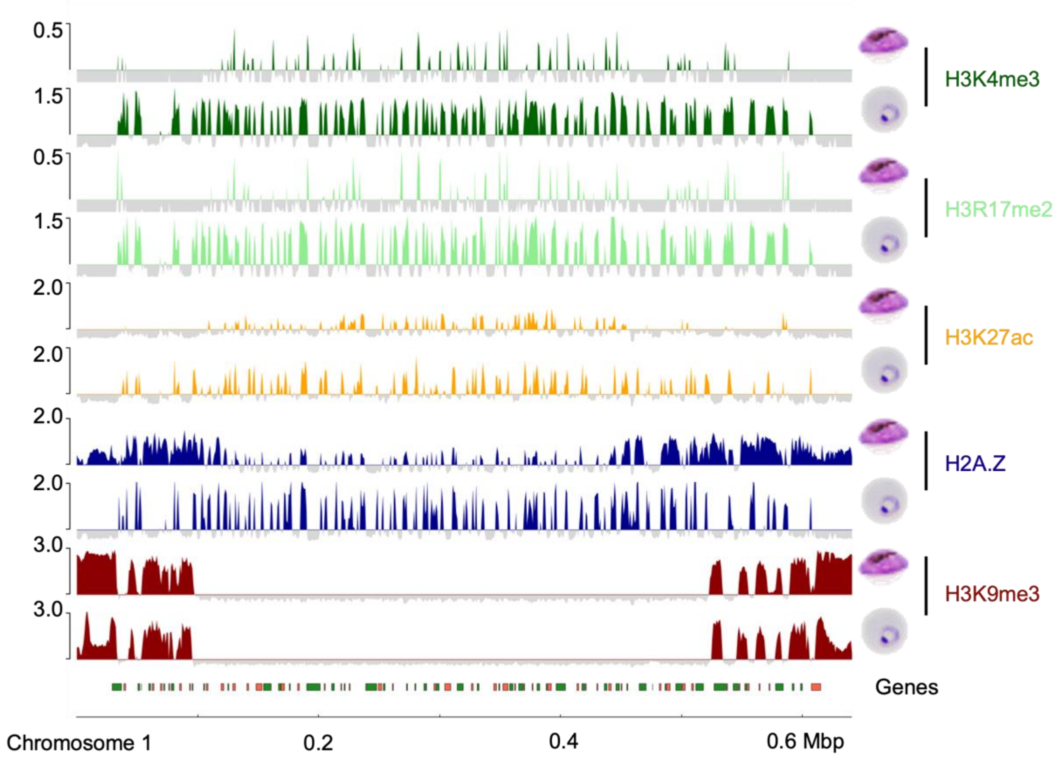
0.5
1.5
0.5
1.5
2.0
2.0
2.0
2.0
3.0
3.0
H3K4me3
H3R17me2
H3K27ac
H2A.Z
H3K9me3
Genes
Chromosome 1
0.2
0.4
0.6 Mbp

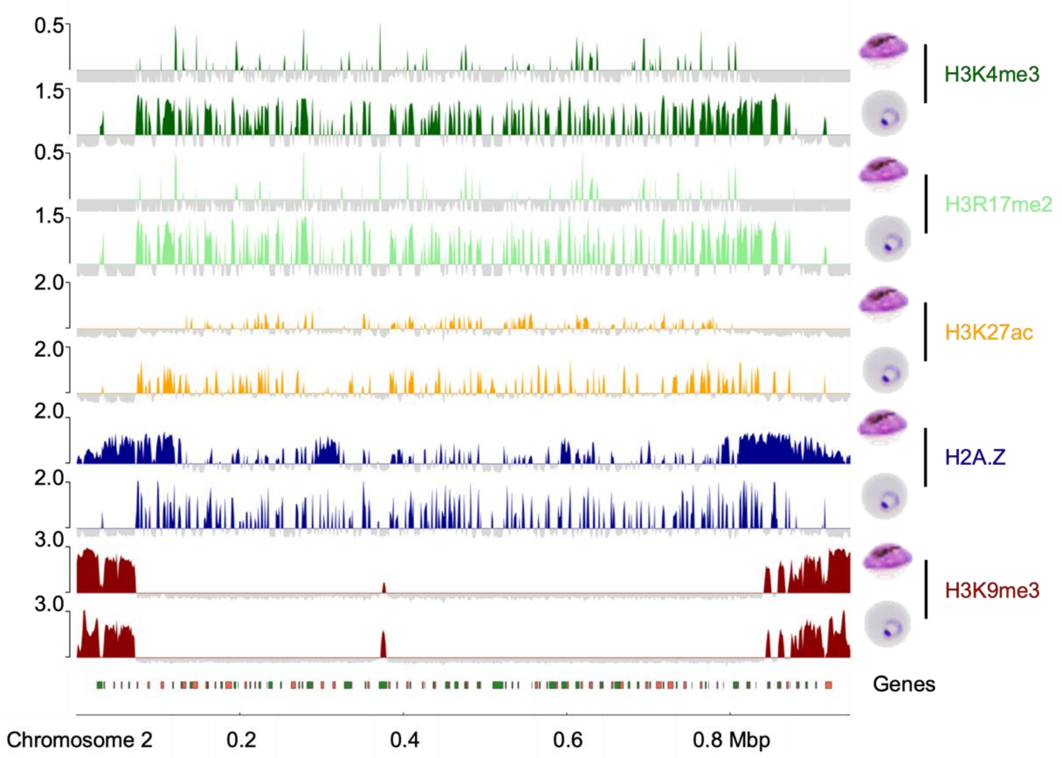
0.5
1.5
0.5
1.5
2.0
2.0
2.0
2.0
3.0
3.0
H3K4me3
H3R17me2
H3K27ac
H2A.Z
H3K9me3
Genes
Chromosome 2
0.2
0.4
0.6
0.8 Mbp

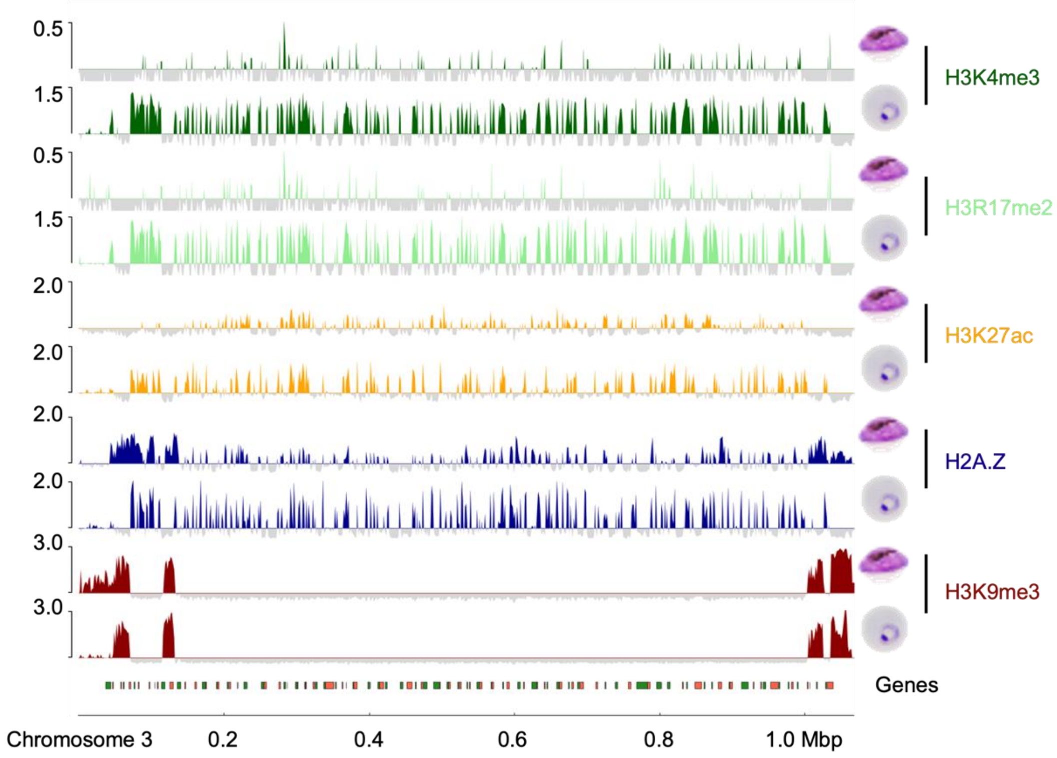

0.5
1.5
0.5
1.5
2.0
2.0
2.0
2.0
3.0
3.0
H3K4me3
H3R17me2
H3K27ac
H2A.Z
H3K9me3
Genes
Chromosome 3
0.2
0.4
0.6
0.8
1.0 Mbp

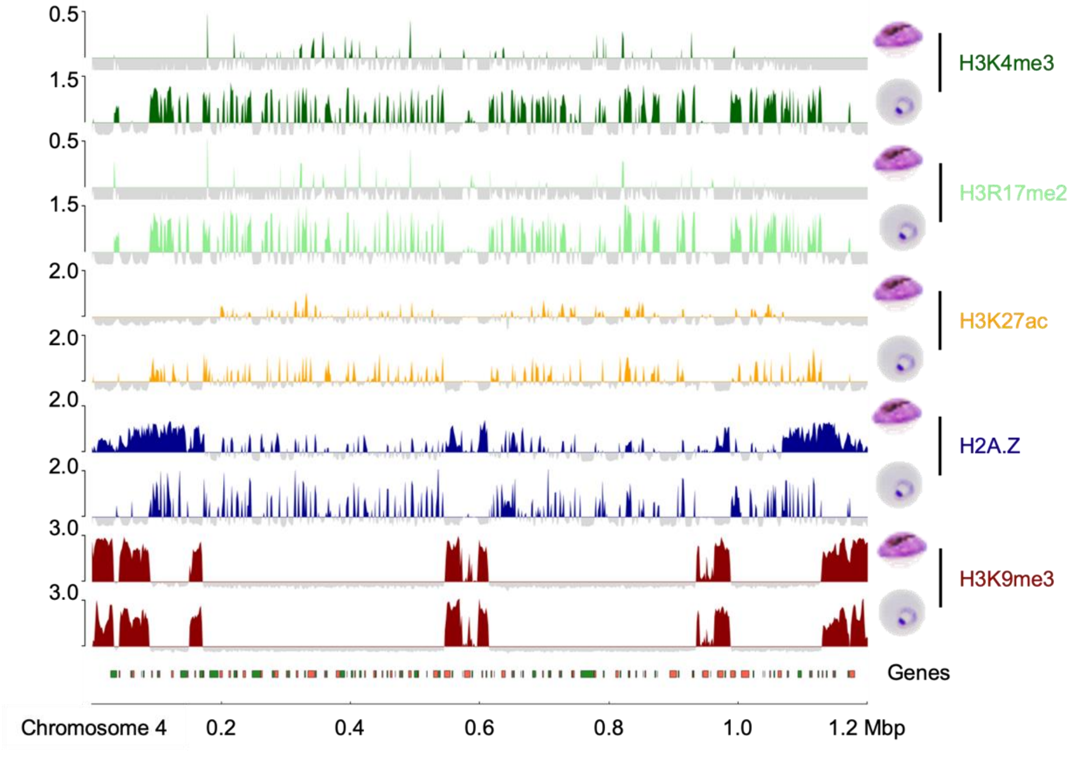

0.5
1.5
0.5
1.5
2.0
2.0
2.0
2.0
3.0
3.0
H3K4me3
H3R17me2
H3K27ac
H2A.Z
H3K9me3
Genes
Chromosome 4
0.2
0.4
0.6
0.8
1.0
1.2 Mbp

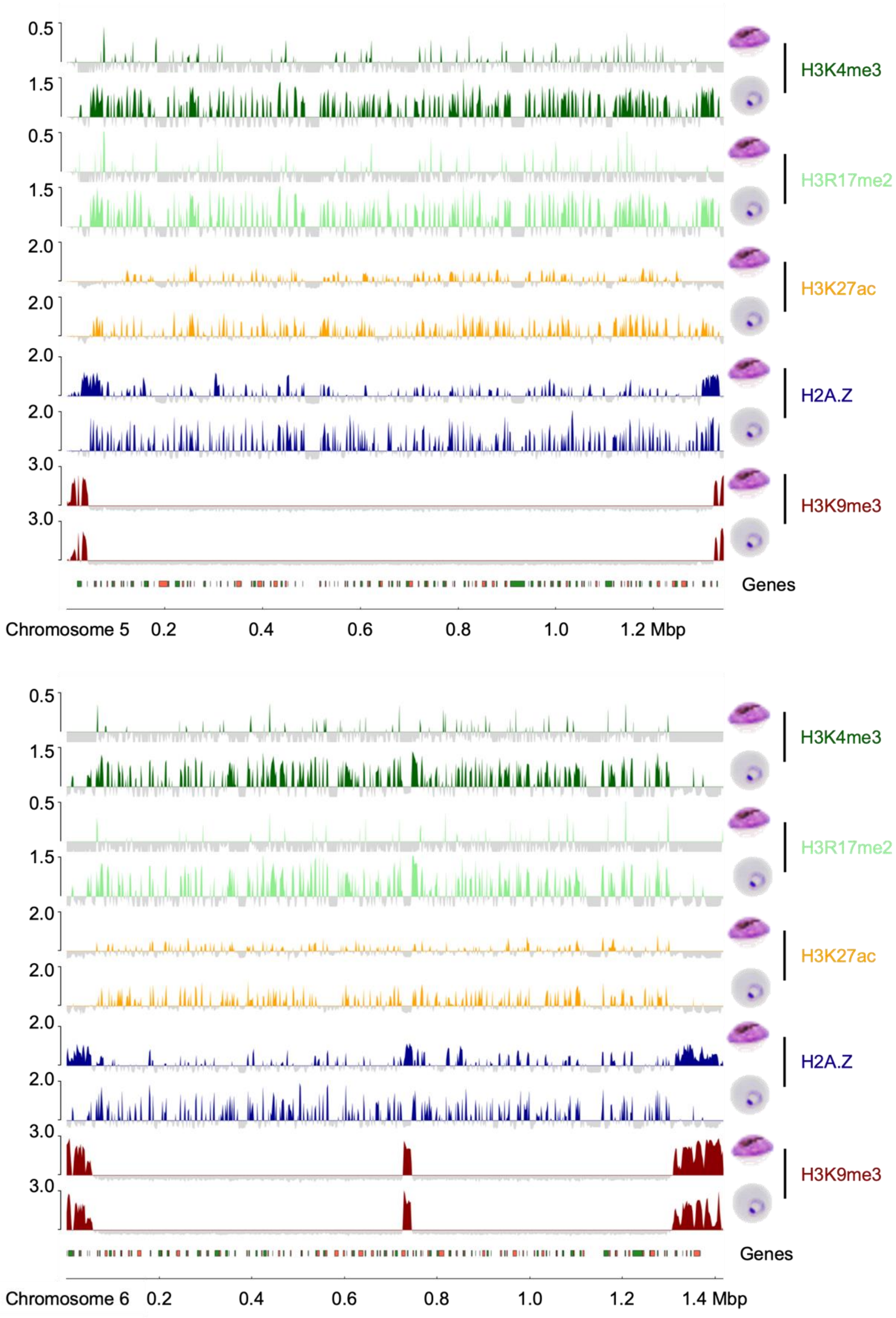
0.5
1.5
0.5
1.5
2.0
2.0
2.0
2.0
3.0
3.0
H3K4me3
H3R17me2
H3K27ac
H2A.Z
H3K9me3
Genes
Chromosome 5
0.2
0.4
0.6
0.8
1.0
1.2 Mbp
0.5
1.5
0.5
1.5
2.0
2.0
2.0
2.0
3.0
3.0
H3K4me3
H3R17me2
H3K27ac
H2A.Z
H3K9me3
Genes
Chromosome 6
0.2
0.4
0.6
0.8
1.0
1.2
1.4 Mbp

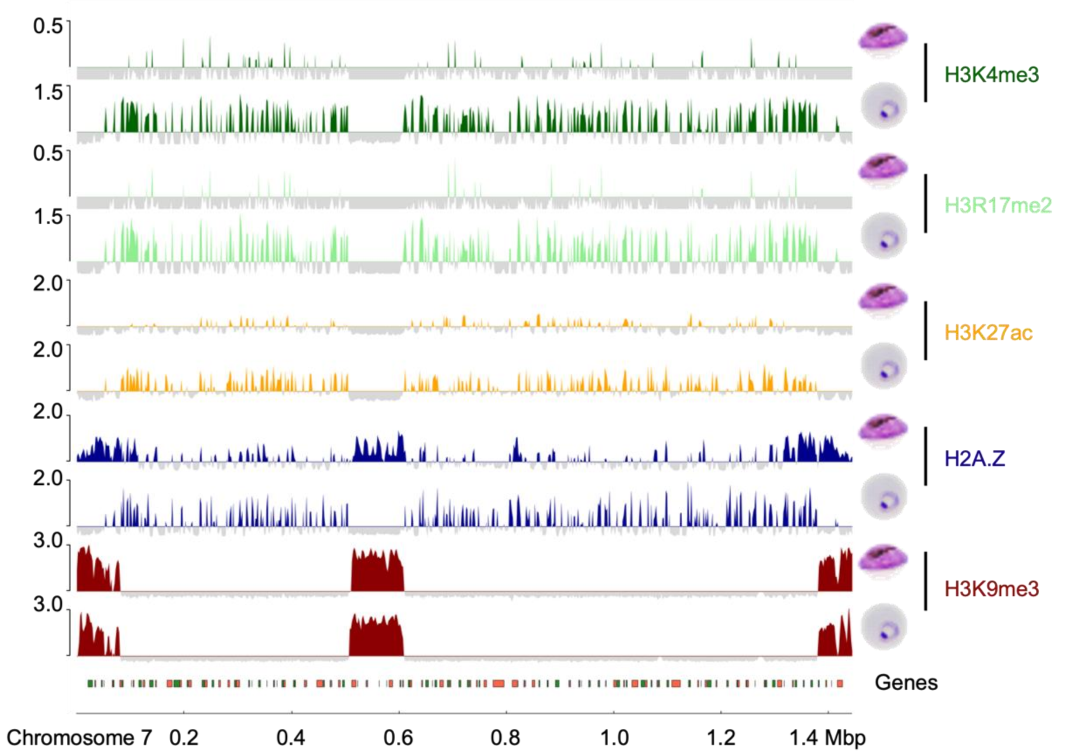
0.5
1.5
0.5
1.5
2.0
2.0
2.0
2.0
3.0
3.0
H3K4me3
H3R17me2
H3K27ac
H2A.Z
H3K9me3
Genes
Chromosome 7
0.2
0.4
0.6
0.8
1.0
1.2
1.4 Mbp

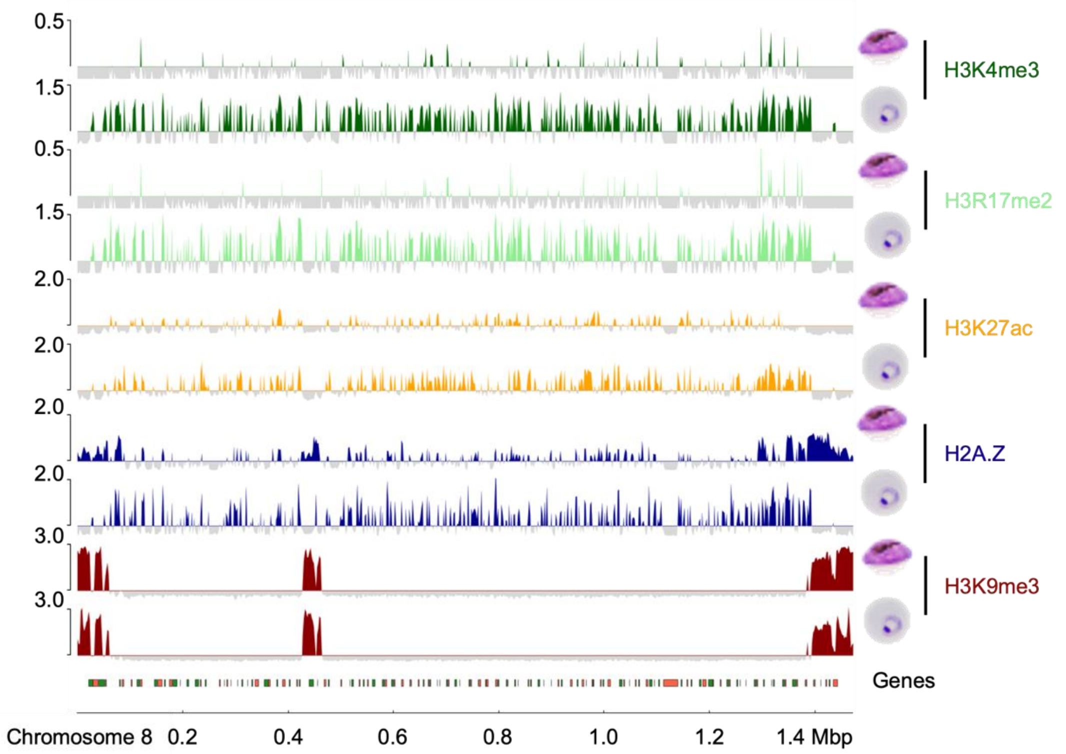
0.5
1.5
0.5
1.5
2.0
2.0
2.0
2.0
3.0
3.0
H3K4me3
H3R17me2
H3K27ac
H2A.Z
H3K9me3
Genes
Chromosome 8
0.2
0.4
0.6
0.8
1.0
1.2
1.4 Mbp

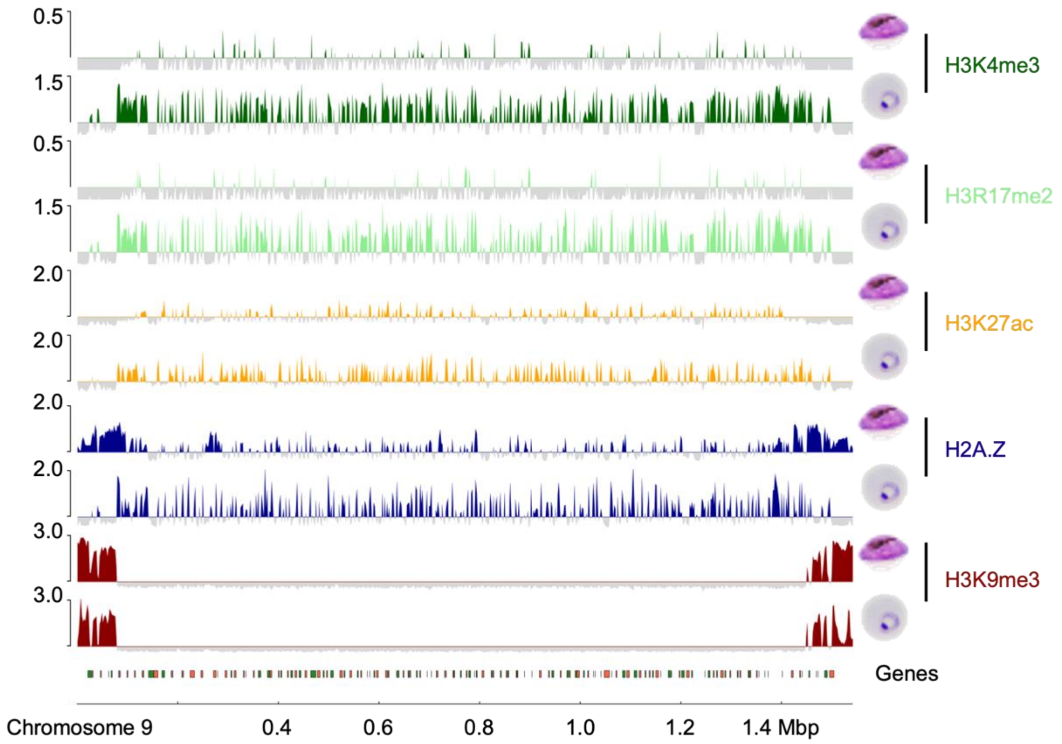
0.5
1.5
0.5
1.5
2.0
2.0
2.0
2.0
3.0
3.0
H3K4me3
H3R17me2
H3K27ac
H2A.Z
H3K9me3
Genes
Chromosome 9
0.4
0.6
0.8
1.0
1.2
1.4 Mbp

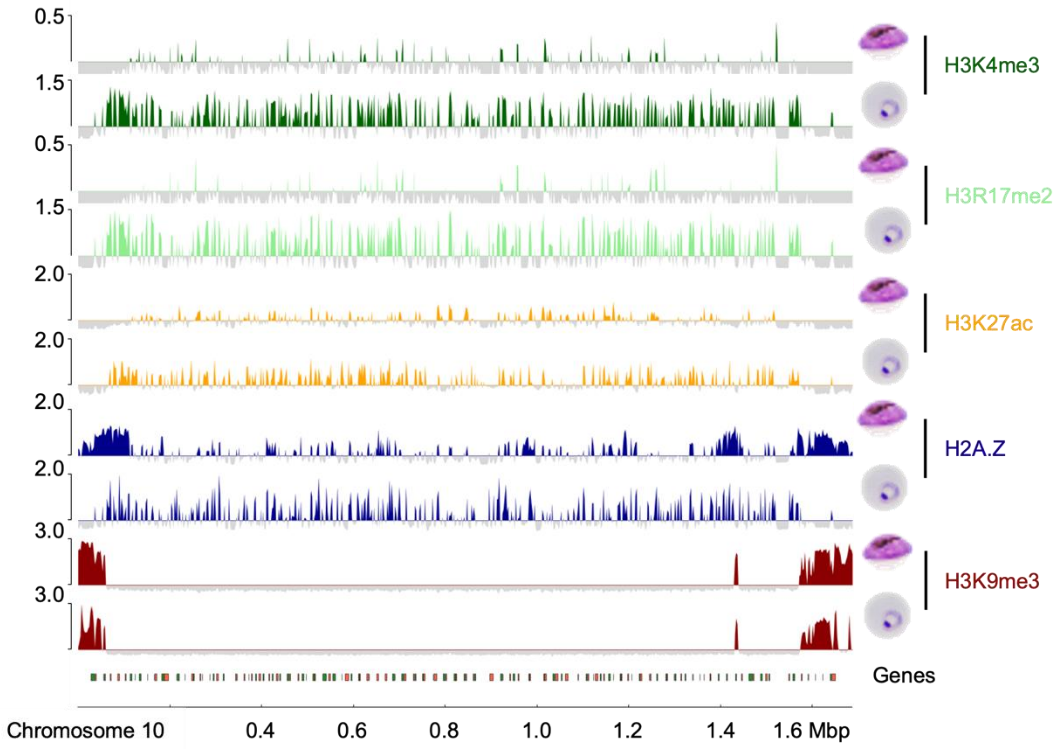
0.5
1.5
0.5
1.5
2.0
2.0
2.0
2.0
3.0
3.0
H3K4me3
H3R17me2
H3K27ac
H2A.Z
H3K9me3
Genes
Chromosome 10
0.4
0.6
0.8
1.0
1.2
1.4
1.6 Mbp

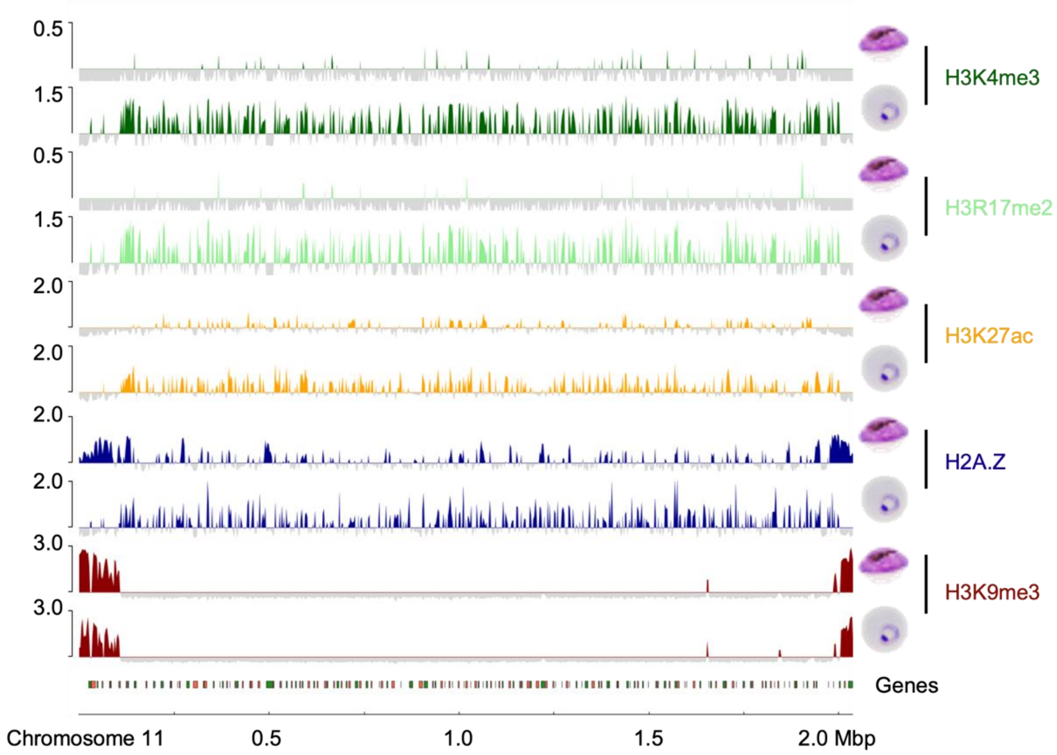
0.5
1.5
0.5
1.5
2.0
2.0
2.0
2.0
3.0
3.0
H3K4me3
H3R17me2
H3K27ac
H2A.Z
H3K9me3
Genes
Chromosome 11
0.5
1.0
1.5
2.0 Mbp

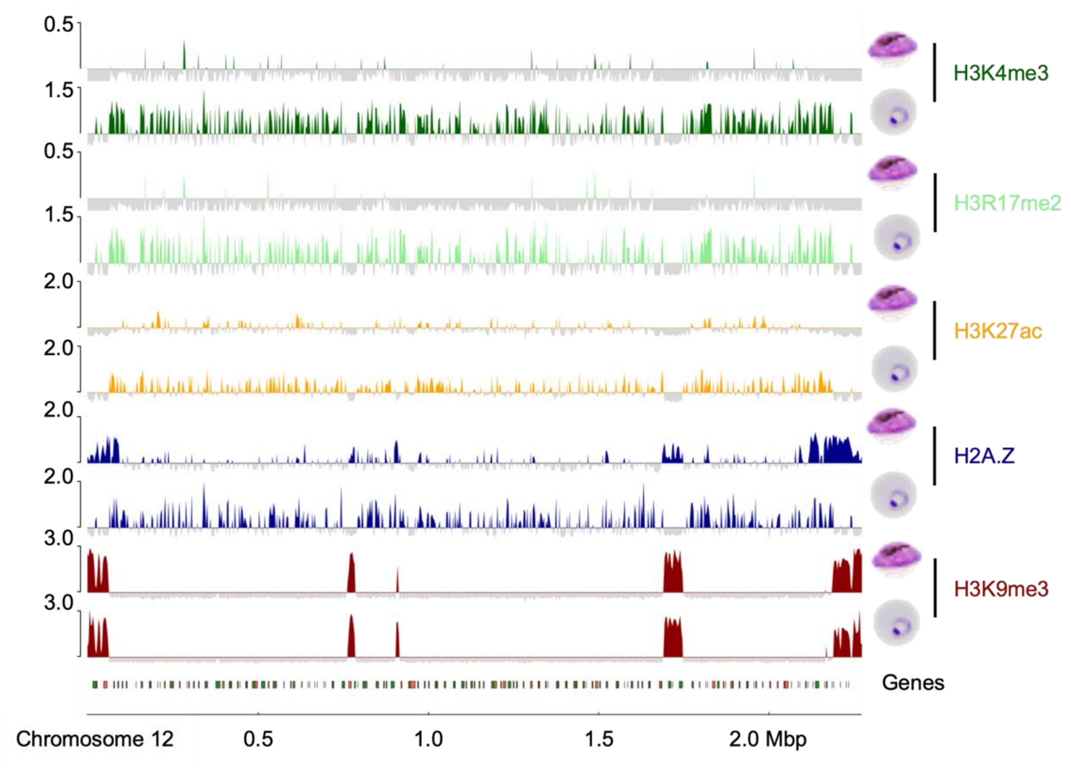
0.5
1.5
0.5
1.5
2.0
2.0
2.0
2.0
3.0
3.0
H3K4me3
H3R17me2
H3K27ac
H2A.Z
H3K9me3
Genes
Chromosome 12
0.5
1.0
1.5
2.0 Mbp

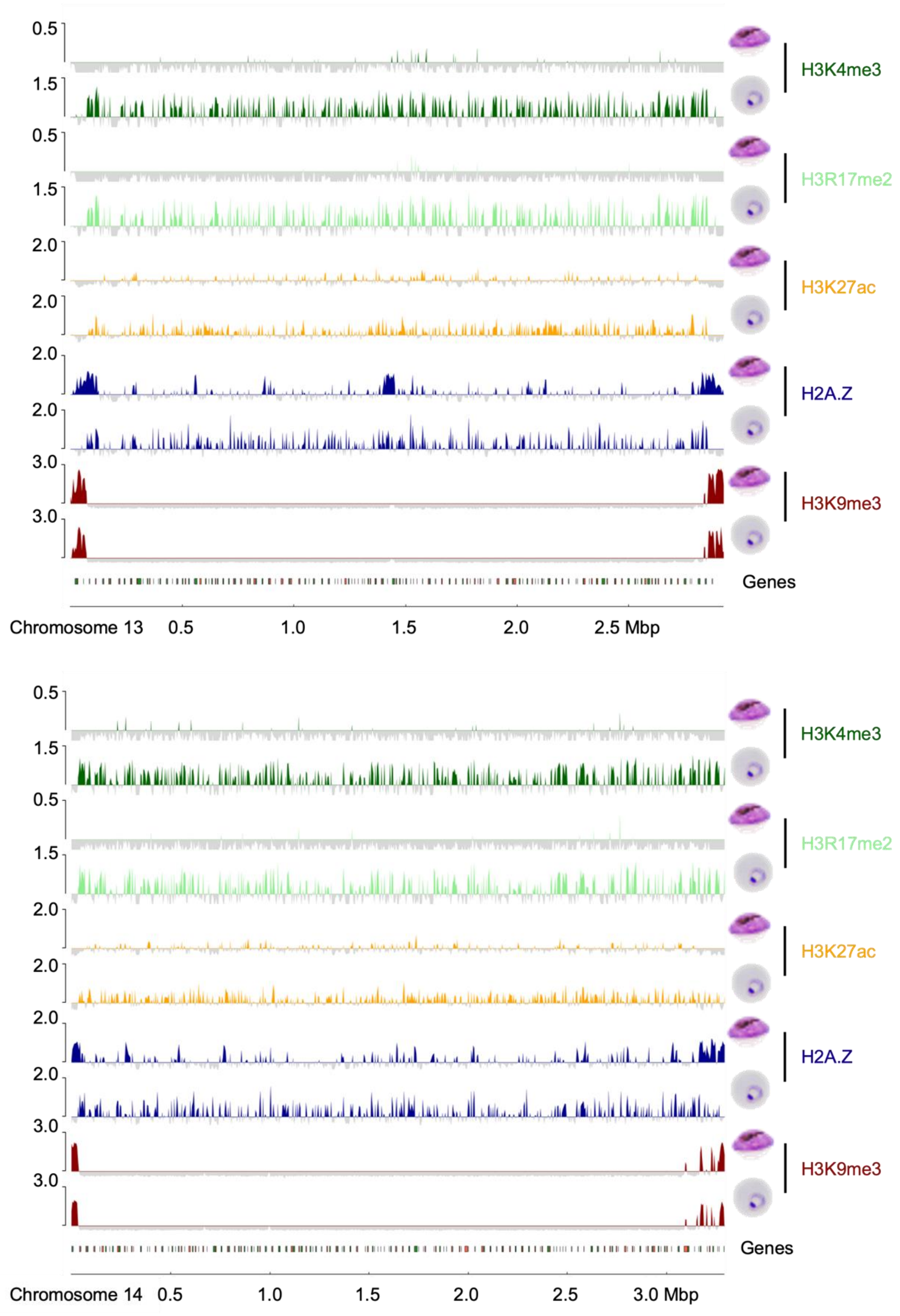

Figure *S*3: Overview over log_2-transformed ChIP/Input coverage across all 14 *P. falciparum* chromosomes in female gametocytes and ring stage parasites. H3K4me3 (n=2 for female gametocytes and rings), H3R17me2 (n=2 for female gametocytes and rings), H3K27ac (n=3 for female gametocytes, n=2 for rings), H2A.Z (n=6 for female gametocytes, n=2 for rings), H3K9me3 (n=5 for female gametocytes, n=2 for rings).

Figure S4

A

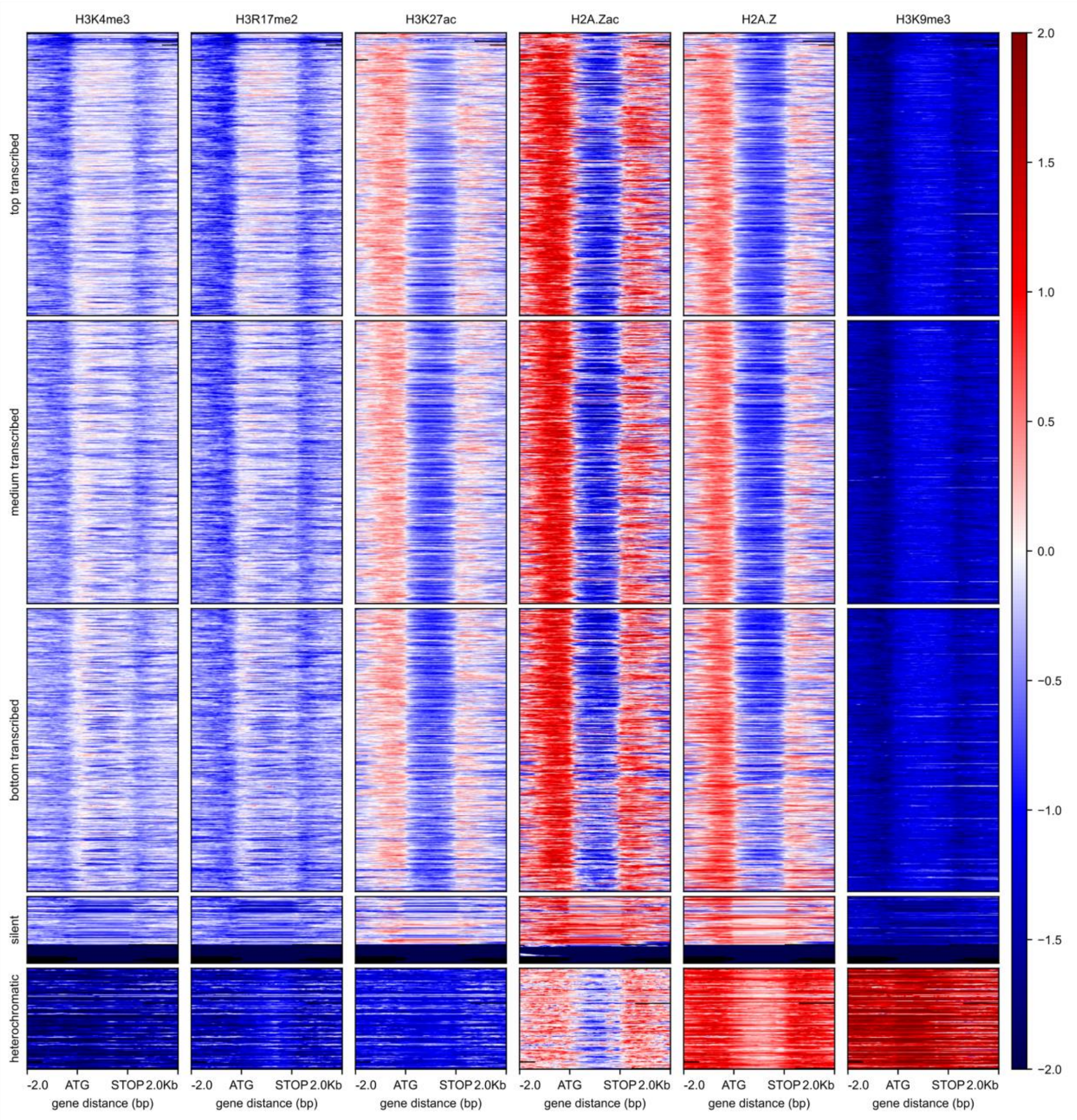

H3K4me3
H3R17me2
H3K27ac
H2A.Zac
H2A.Z
H3K9me3
top transcribed
medium transcribed
bottom transcribed
silent
heterochromatic
-2.0
ATG
STOP
2.0Kb
gene distance (bp)
2.0
1.5
1.0
0.5
0.0
−0.5
−1.0
−1.5
−2.0

B

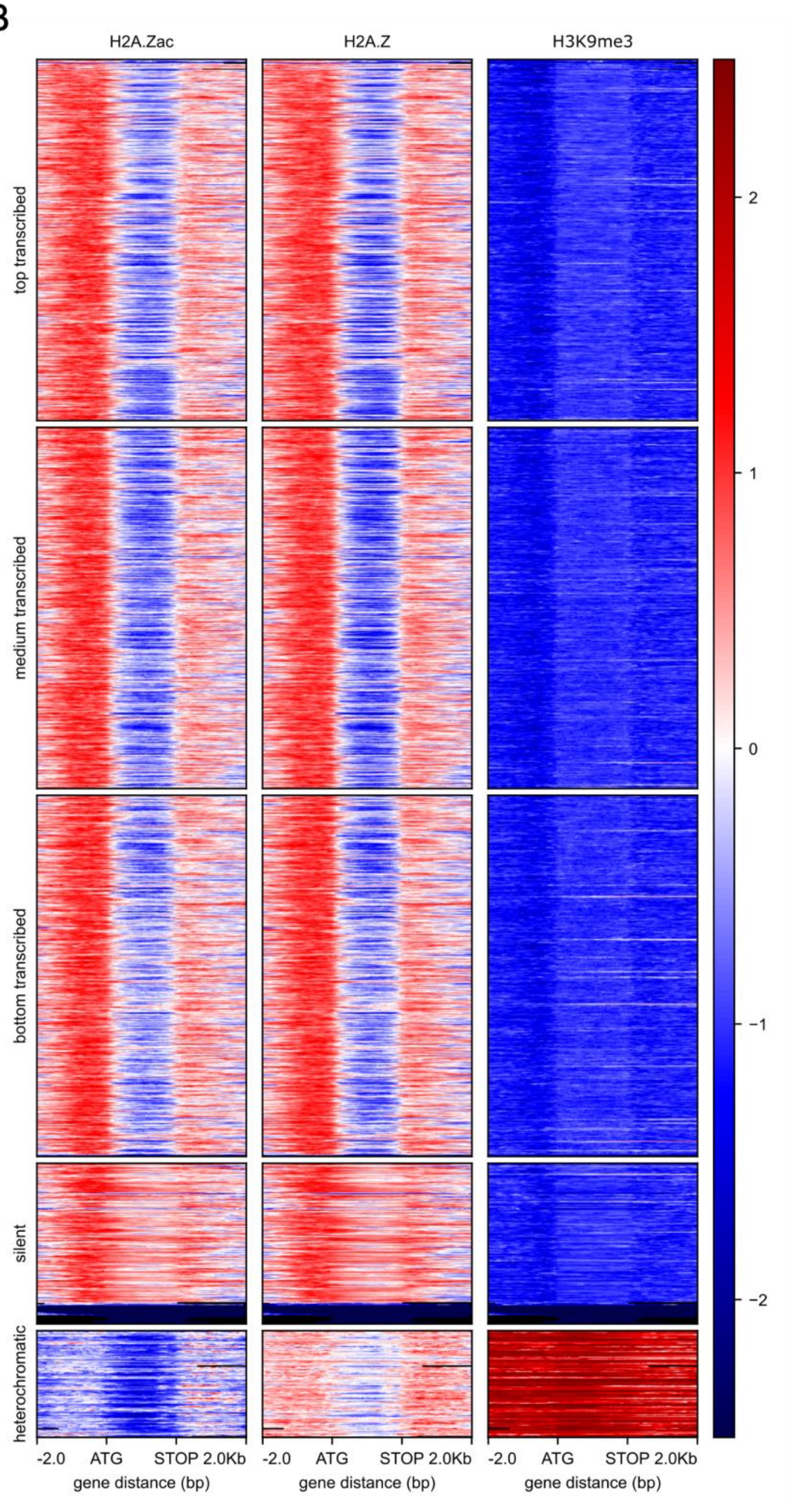

H2A.Zac
H2A.Z
H3K9me3
top transcribed
medium transcribed
bottom transcribed
silent
heterochromatic
2
1
0
−1
−2
-2.0
ATG
STOP 2.0Kb
gene distance (bp)

C

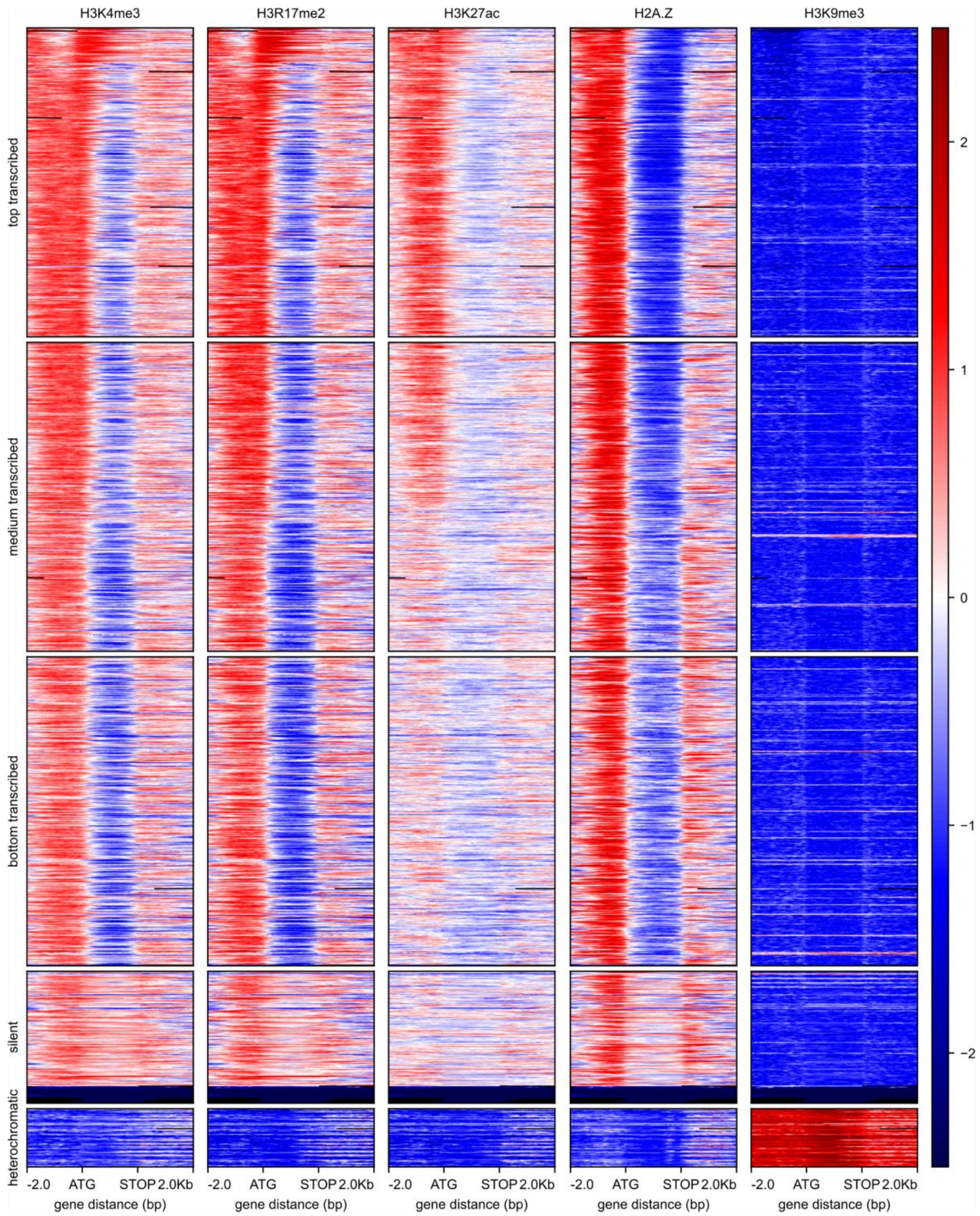

Figure S4: Heatmaps showing the log_2-transformed ChIP/Input coverage of all genes and their 2 kb up/downstream region for each modification sorted into groups according to their expression. The black bar in the silent genes represents silent mitochondrial and apicoplast genes that carry no histone modifications. A) Day 6 female gametocytes. B) Day 6 male gametocytes. C) Ring stage parasites. H3K4me3 (n=2 for female gametocytes and rings), H3R17me2 (n=2 for female gametocytes and rings), H3K27ac (n=3 for female gametocytes, n=2 for rings), H2A.Z (n=6 for female

gametocytes, n=2 for male gametocytes, n=2 for rings), H2A.Zac (n=2 for female gametocytes, n=1 for male gametocytes), H3K9me3 (n=5 for female gametocytes, n=2 for male gametocytes, n=2 for rings).

Figure S5

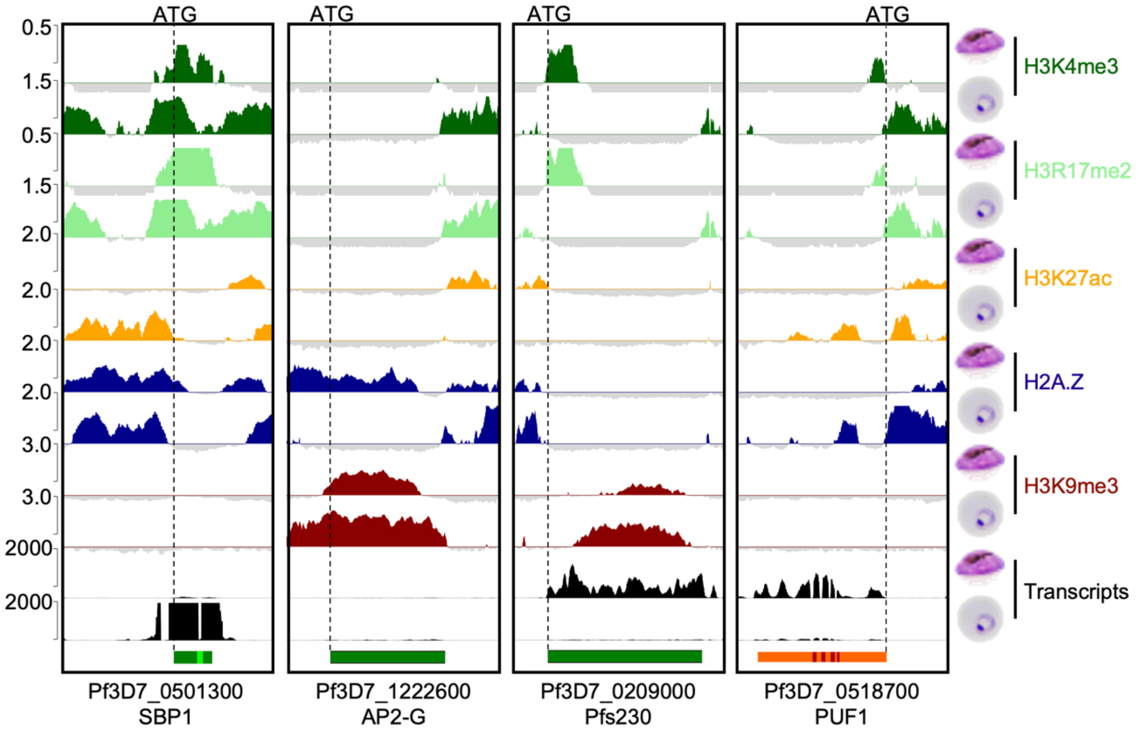

Figure S5: Global ChIPseq and RNAseq profiles for regions of particular interest in female gametocytes and ring stage parasites. Genes are marked depending on their genomic orientation in either green (+ genes) or orange (- genes). Introns are shaded in light green or dark red. The position of the ATG is marked by a dashed line. Transcripts (FPKM) are shown in black. Regions shown: SBP1 from 65.5 kbp to 72 kbp, AP2-G from 904.7 kbp to 918 kbp, Pfs230 from 368.4 kbp to 381.2 kbp, PUF1 from 773.2 kbp to 783.5 kbp. H3K4me3 (n=2 for female gametocytes and ring stage parasites), H3R17me2 (n=2 for female gametocytes and ring stage parasites), H3K27ac (n=3 for female gametocytes, n=2 for ring stage parasites), H2A.Z (n=6 for female gametocytes, n=2 for ring stage parasites), H3K9me3 (n=5 for female gametocytes, n=2 for ring stage parasites).

Figure S6

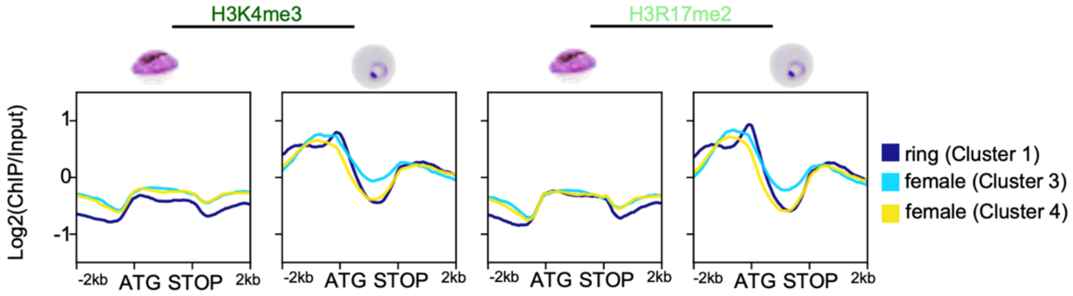

Figure S6: H3K4me3 and H3R17me2 coverage in genes with peak expression in asexual or sexual parasites. Average log2-transformed ChIP/Input coverage plots from two biological replicates for H3K4me3 and H3R17me2 in day 6 female gametocytes and ring stage parasites over genes with peak expression in early (cluster 3) and late (cluster 4) female gametocytes and rings (cluster 1).

Figure S7

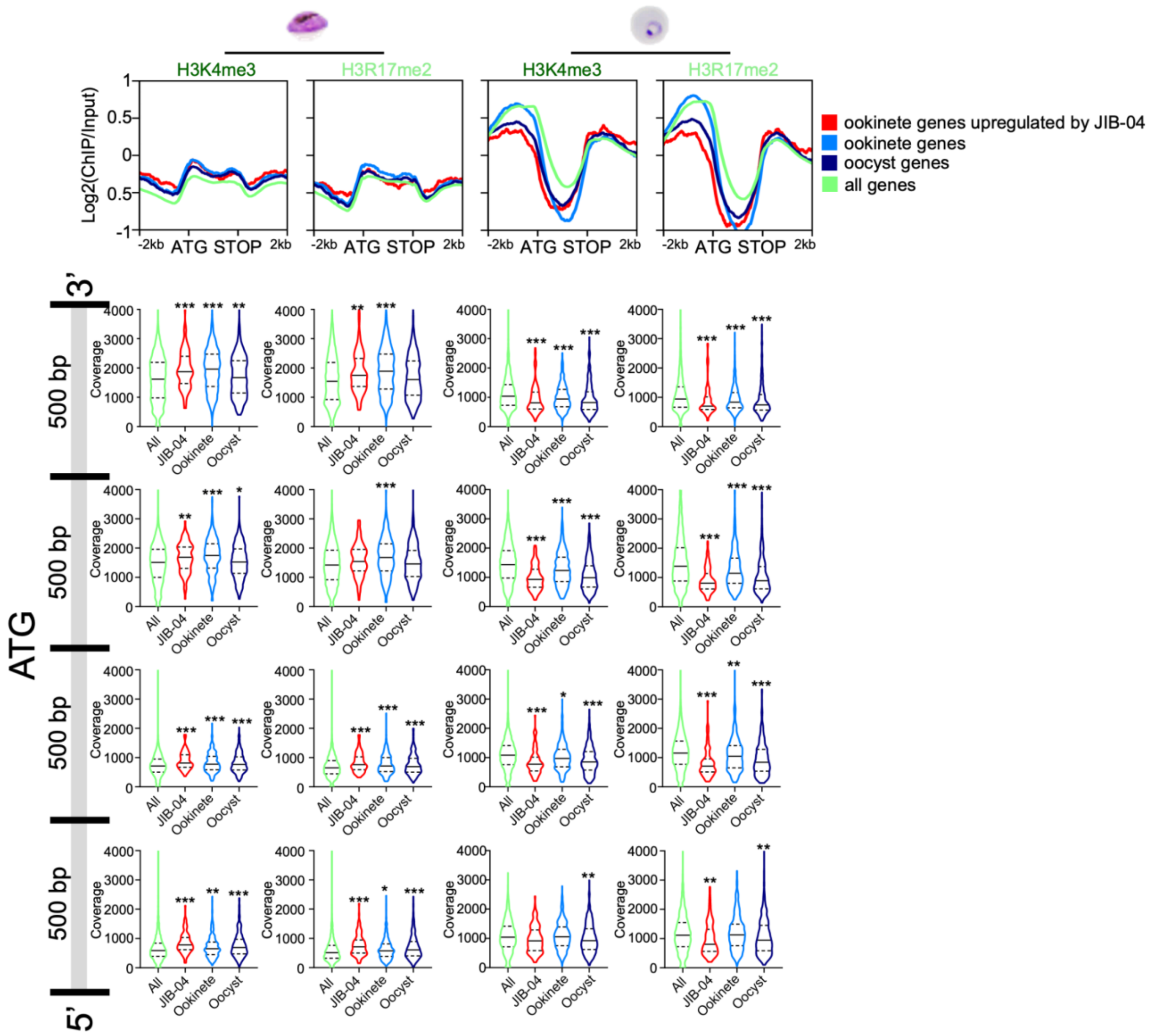

Figure S7: H3K4me3/H3R17me2 coverage in gametocytes is significantly increased in ookinete and oocyst specific genes. Log_2-transformed ChIP/Input coverage plots from two biological replicates for H3K4me3 and H3R17me2 in day 6 female gametocytes and ring stage parasites over ookinete genes upregulated after JIB-04 treatment ([18], available as Supplementary Table), ookinete genes ([48], available on PlasmoDB) and oocyst genes ([77], available on PlasmoDB) compared to all genes. Plotted is the cumulative coverage over the indicated regions for each gene group. Significant differences between the average coverage of all genes and the specific gene groups were determined by one-way ANOVA and are indicated with stars over the bars, * $p < 0.05$, ** $p < 0.01$, *** $p < 0.001$.

Figure S8

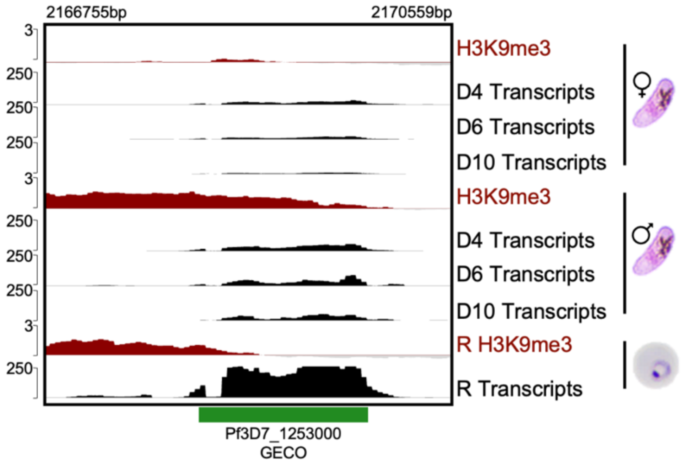

Figure S8: Heterochromatin and transcription profile of GECO (gametocyte erythrocyte cytosolic protein), region shown from 2167 kbp to 2170 kbp. RNAseq data: Male gametocytes: day 4: n=3, day 6: n=2, day 10: n=1; female gametocytes: day 4: n=2, day 6: n=4, day 10: n=4; ring stage parasites: n=2; ChIPseq data: H3K9me3 (n=5 for female gametocytes, n=2 for male gametocytes, n=2 for ring stage parasites).

Figure S9

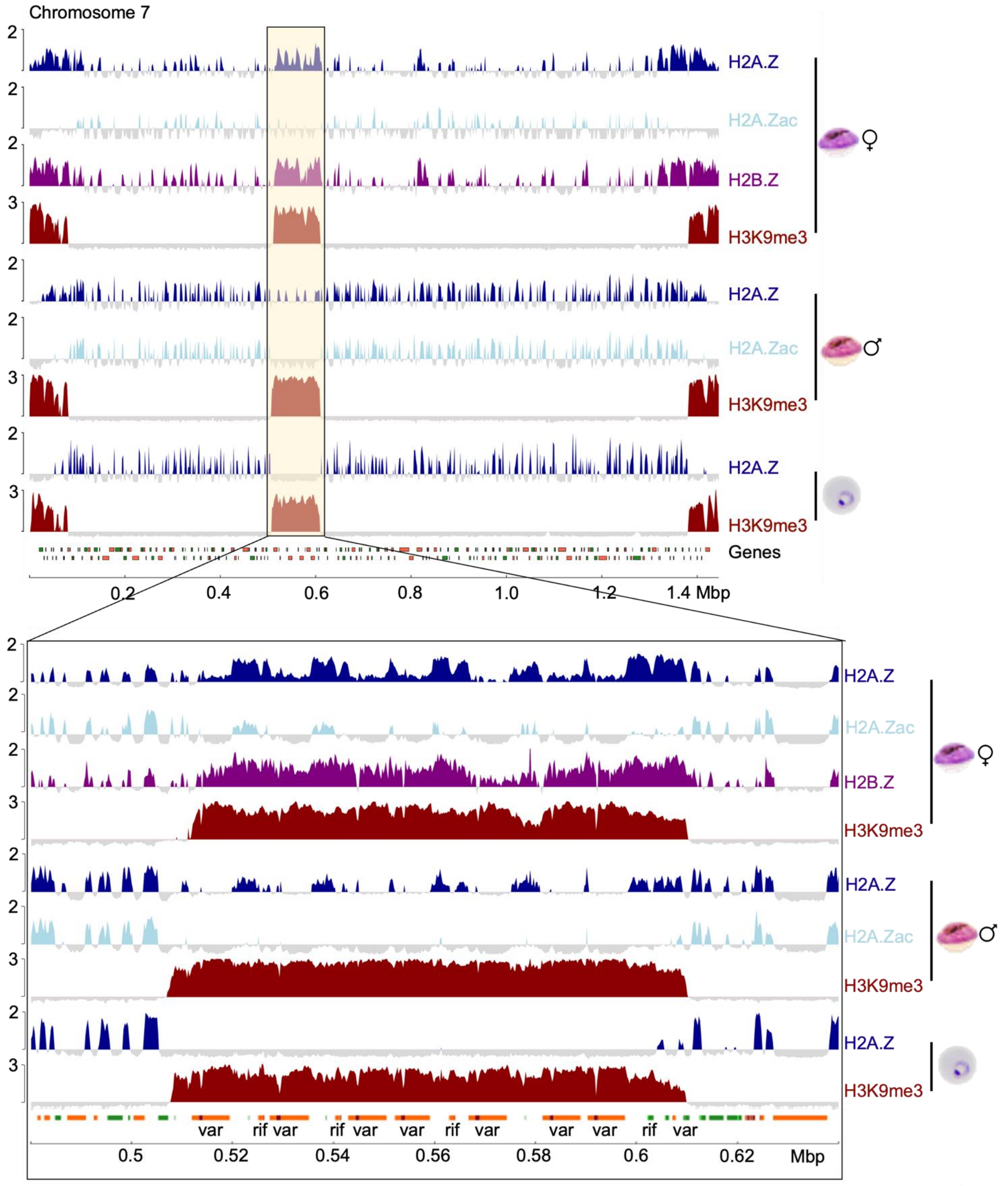

Figure S9: H2A.Z is associated with heterochromatic areas in day 6 female gametocytes. Overview over chromosome 7 showing log_2-transformed ChIP/Input coverage for H2A.Z (n=6 for female gametocytes, n=2 for male gametocytes, n=2 for ring stage parasites) and H3K9me3 (n=5 for

female gametocytes, n=2 for male gametocytes, n=2 for ring stage parasites) in all stages, H2A.Zac in male (n=1) and female gametocytes (n=2), and H2B.Z in female gametocytes (n=1). Zoom into internal heterochromatic cluster on chromosome 7. Genes are marked in either green (+ genes) or orange (- genes). Introns are marked either in light green or dark red, depending on gene orientation.

Figure S10

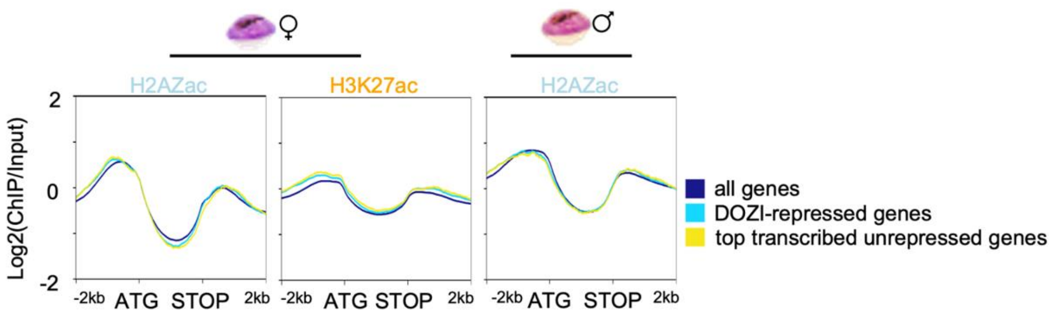

Figure S10: H3K27ac and H2A.Zac coverage is unchanged in translationally repressed genes. Average log2-transformed ChIP/Input coverage plots for H2A.Zac (n=2 for female gametocytes, n=1 for male gametocytes) in day 6 male and female gametocytes and H3K27ac in female gametocytes (n=3) over all genes in comparison to genes ranking in the top expressed tercile (excluding DOZI-repressed genes) and DOZI-repressed genes in day 6 female gametocytes.

Figure S11

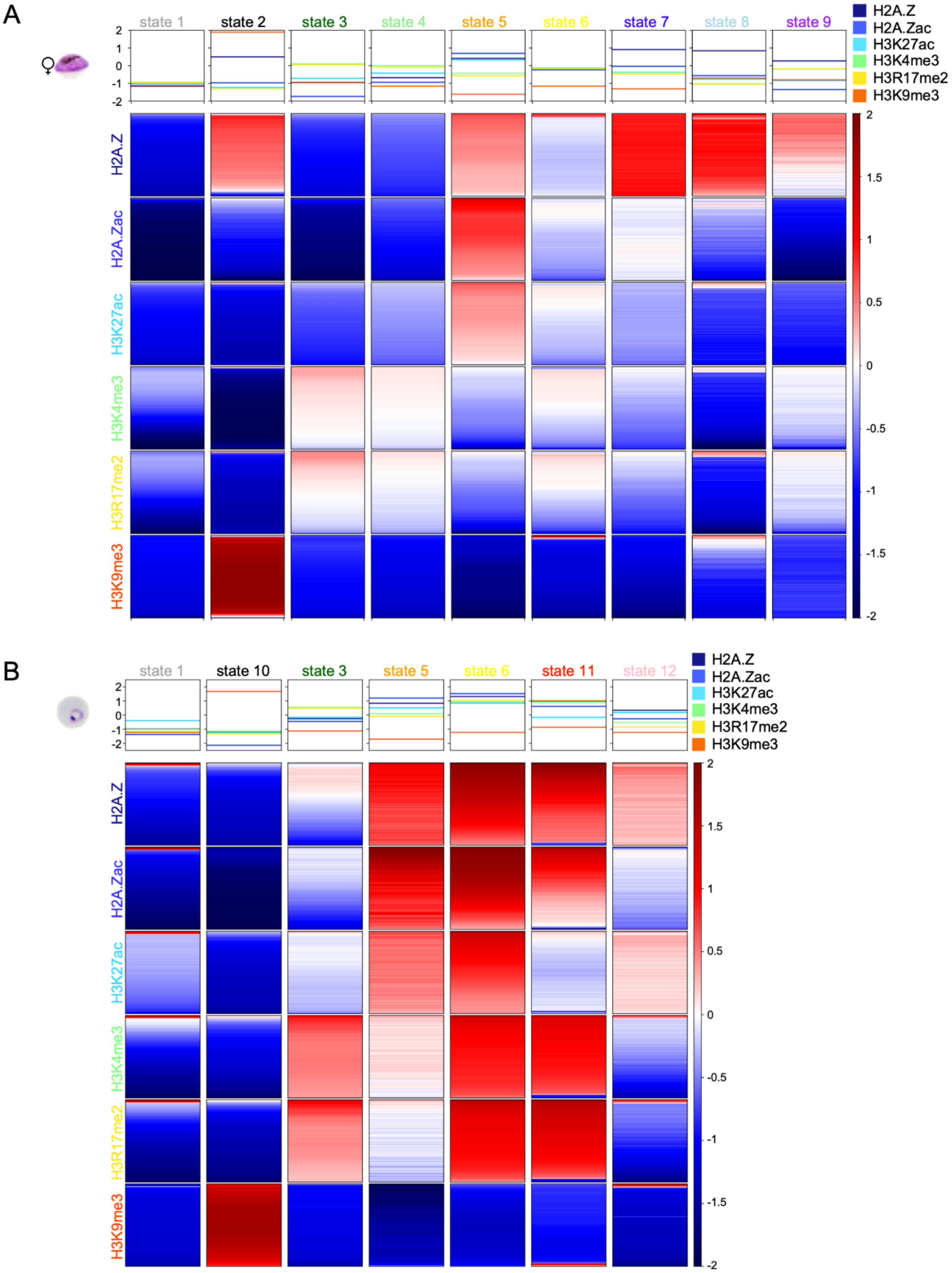

Figure S11: Heat maps of chromatin states discovered in female day 6 gametocyte and ring stage parasites. Heatmap showing the log_2-transformed ChIP/Input coverage of the studied histone modifications over all 50 bp bins represented in each of the A) female day 6 gametocyte states and B) ring stage parasite states.

Figure S12

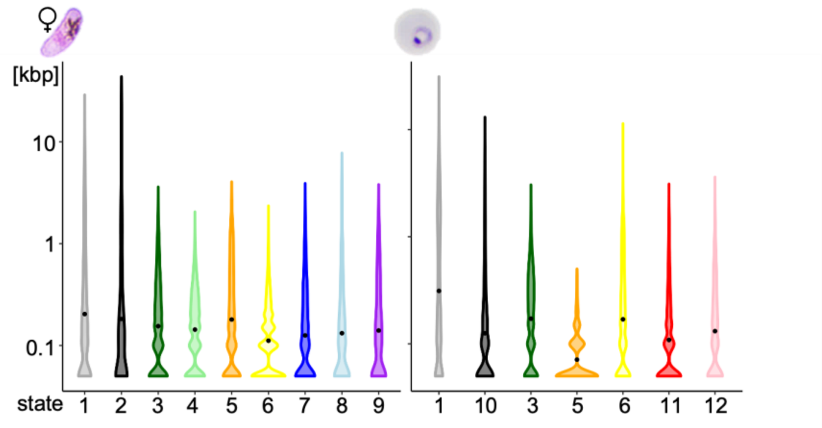

Figure S12: Length distribution of chromatin states. Neighbouring bins with identical chromatin states were fused and the lengths of the continuous regions with the same chromatin state were plotted as violin plots for female day 6 gametocytes and ring stage parasites.
