# Supplementary material for: Plasmodium falciparum gametocytes display global chromatin remodelling during sexual differentiation
Source: BMC Biol. 2023 Apr 3;21:65. doi: 10.1186/s12915-023-01568-4 (PMC10071754; doi:10.1186/s12915-023-01568-4)
Supplement: Supplementary file 6 — Additional file 6: Table S5. Antibodies used for western blot (WB), immunofluorescence analysis (IFA), and chromatin immunoprecipitation (ChIP). [file 12915_2023_1568_MOESM6_ESM.docx]

**Table S5: Antibodies used for western blot (WB), immunofluorescence analysis (IFA), and chromatin immunoprecipitation (ChIP)**

| Target | Species | Reference | Dilution factor / Concentration | | |
| --- | --- | --- | --- | --- | --- |
|  |  |  | IFA | WB | ChIP |
| H2A.Z | Rabbit | Petter et al. 2011 | - | 1:2500 | 1:167 |
| H2A.Zac | Rabbit | custom^1)^, Anaspec | - | 1:2500 | 1:167 |
| H2B.Z | Rabbit | custom^2)^, Anaspec | - | 1:2500 | 1:167 |
| H3K9me3 | Rabbit | Active Motif 39161 | - | 1:2500 | 1:167 |
| HP1 | Rabbit | Petter et al. 2011 | - | 1:2500 | 1:167 |
| H3K4me3 | Rabbit | Abcam ab8580 | - | 1:2500 | 1:167 |
| H3R17me2 | Rabbit | Abcam ab8284 | - | 1:2500 | 1:167 |
| H3K27ac | Rabbit | Abcam ab4729 | - | 1:2500 | 1:167 |
| H3 | Rabbit | Abcam ab1791 | - | 1:5000 | - |
| Aldolase | Rabbit | Abcam ab207494 | - | 1:10.000 | - |
| GFP | Rabbit | Thermo Fisher Scientific A6455 | 1:500 | - | - |
| Rabbit-Alexa 488 | Goat | Life Tech A-11034 | 1:1000 | - | - |
| Rabbit-HRP | Goat | Invitrogen A16110 | - | 1:10.000 | - |
| Hoechst 33342 | - | Thermo Fisher Scientific 62249 | 200 nM | - | - |

1) Peptides from PfH2A.Z residues 6-18, acetylated on lysine 11 and 15 were synthesised, conjugated to keyhole limpet hemocyanin (KLH) and used to immunise rabbits (Anaspec). The serum was then affinity purified against the acetylated peptide and cross-adsorbed against non-acetylated peptide (Azizan et al., unpublished).

2) Peptides from PfH2B.Z residues 1-20, acetylated on lysine 3 and 8 were synthesised, conjugated to keyhole limpet hemocyanin (KLH) and used to immunise rabbits (Anaspec). The serum was then affinity purified against the acetylated and non-acetylated forms of the peptide (Azizan et al., unpublished).
