# Supplementary material for: Plasmodium falciparum gametocytes display global chromatin remodelling during sexual differentiation
Source: BMC Biol. 2023 Apr 3;21:65. doi: 10.1186/s12915-023-01568-4 (PMC10071754; doi:10.1186/s12915-023-01568-4)
Supplement: Supplementary file 7 — Additional file 7. Uncropped western blots shown in Figure S2. [file 12915_2023_1568_MOESM7_ESM.pdf]

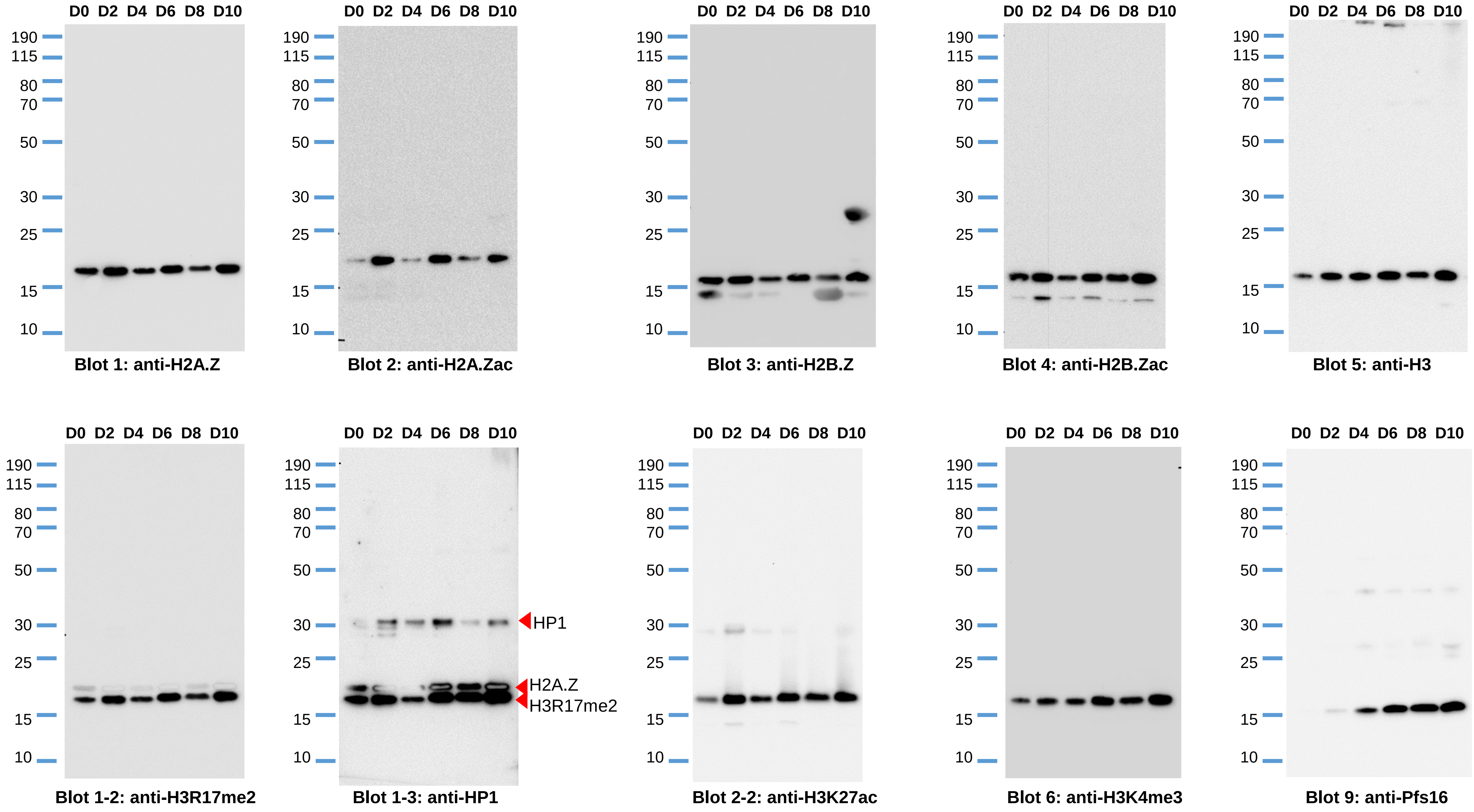

Additional File 7: Uncropped western blots shown in Figure S2. Blot 1 was incubated consecutively with anti-H2A.Z, anti-H3R17me2 and anti-HP1. Blot 2 was incubated consecutively with anti-H2A.Zac and anti-H3K27ac. The different histone modifications can be discriminated based on their different molecular weights.
